# Community-based models of care facilitating the recovery of people living with persistent and complex mental health needs: a systematic review and narrative synthesis

**DOI:** 10.3389/fpsyt.2023.1259944

**Published:** 2023-09-14

**Authors:** Carol Harvey, Tessa-May Zirnsak, Catherine Brasier, Priscilla Ennals, Justine Fletcher, Bridget Hamilton, Helen Killaspy, Peter McKenzie, Hamilton Kennedy, Lisa Brophy

**Affiliations:** ^1^Department of Psychiatry, University of Melbourne, Melbourne, VIC, Australia; ^2^North West Area Mental Health, Division of Mental Health, Northern Health, Melbourne, VIC, Australia; ^3^Social Work and Social Policy, Department of Community and Clinical Health, La Trobe University, Bundoora, VIC, Australia; ^4^Neami National, Preston, VIC, Australia; ^5^Centre for Mental Health, Melbourne School of Population and Global Health, University of Melbourne, Melbourne, VIC, Australia; ^6^Centre for Mental Health Nursing, Department of Nursing, The University of Melbourne, Melbourne, VIC, Australia; ^7^Department of Epidemiology and Applied Clinical Research, Division of Psychiatry, University College London, London, United Kingdom; ^8^Camden and Islington NHS Foundation Trust, London, United Kingdom; ^9^The Bouverie Centre, School of Psychology and Public Health, La Trobe University, Brunswick, VIC, Australia

**Keywords:** recovery, complex, mental illness, models of care, community

## Abstract

**Objective:**

This study aims to assess the effectiveness of community-based models of care (MoCs) supporting the recovery of individuals who experience persistent and complex mental health needs.

**Method:**

We conducted a systematic review and narrative synthesis of MoC studies reporting clinical, functional, or personal recovery from October 2016 to October 2021. Sources were Medline, EMBASE, PsycInfo, CINAHL, and Cochrane databases. Studies were grouped according to MoC features. The narrative synthesis was led by our researchers with lived experience.

**Results:**

Beneficial MoCs ranged from well-established to novel and updated models and those explicitly addressing recovery goals and incorporating peer support: *goal-focused*; *integrated community treatment*; *intensive case management*; *partners in recovery care coordination*; *rehabilitation and recovery-focused*; *social and community connection-focused*; *supported accommodation*; and *vocational support*. None of our diverse group of MoCs supporting recovery warranted a rating of best practice. Established MoCs, such as *intensive case management*, are promising practices regarding clinical and functional recovery, with potential for enhancements to support personal recovery. Emerging practice models that support personal and functional recovery are those where consumer goals and priorities are central.

**Conclusion:**

Evidence for established models of care shows that there is a need for inevitable evolution and adaptation. Considering the high importance of effective MoCs for people experiencing persistent and complex mental health needs, further attention to service innovation and research is required. Greater emphasis on the inclusion of lived and living experience in the design, delivery, implementation, and research of MoCs is needed, to enhance MOCs' relevance for achieving individual consumer recovery outcomes.

## Background

Many people living with severe or persistent mental health conditions experience psychosocial disability and have complex support needs. Although common, the use of diagnosis alone as a proxy for disability is contested (1). Longitudinal evidence suggests that ~25% of people newly diagnosed with a severe mental illness such as schizophrenia, schizoaffective disorder, or bipolar affective disorder develop particularly complex problems and psychosocial disability that may require rehabilitation and/or a multi-sector response (2). The term psychosocial disability (rather than psychiatric disability) recognizes the social determinants and social consequences of disability (3). People living with persistent and complex mental health needs are one of the most excluded groups in society (4).

Thus, a person living with persistent and complex mental health needs may experience difficulties in day-to-day social and occupational functioning and have related needs due to some or all of the following: the impacts of their symptoms or cognitive difficulties; social factors such as homelessness, poverty, unemployment, stigma, and discrimination; and concurrent challenges such as substance use disorders and long-term physical health conditions (5, 6). They are often excluded from participation in education, employment, recreation, and relationships, and from securing stable, safe accommodation (3, 7). Experiences of exclusion may include poor access to health services and for some be compounded by previous negative experiences of the mental health system (including coercion, trauma, and discrimination) which may lead to reluctance to engage with treatment and support (8, 9). Dissatisfaction with treatment and poor recovery outcomes may also be due to limited treatment options, including a lack of access to needed evidence-based psychosocial models of care (10). These may all present significant impediments to both their clinical and personal recovery (11).

Despite considerable needs, this group of people has been missing from recent mental health policy which tends to focus on mental health promotion and the much larger group of people who experience more common mental health issues, such as anxiety and depression (12). For these reasons, we chose to focus this review on people with persistent and complex mental health needs, while noting that most research literature considers a wider group of people, using common but contested descriptors of *serious mental illness* or *severe mental illness*.

The needs and preferences of people living with psychosocial disabilities are diverse, along with their potential for different recovery trajectories. For many people, care and support are required to optimize the potential for a full life in the community (3, 13, 14), so mental health service provision should focus on personal as well as clinical recovery (15). Personal recovery looks different for every person, thwarting simplistic or singular descriptions. It “is ultimately about creating and living a meaningful life in a community of choice, with or without the presence of mental illness” (15, 16). However, the conditions for recovery are increasingly recognized, including empowerment, choice, and meaningful social engagement free of stigma and discrimination. These conditions may, in turn, foster enhanced participation in treatment and support (17).

Mental health services for people living with psychosocial disabilities are configured differently in each country but are typically delivered through clinical and non-government (NGO) sectors. To varying extents, these services focus on enhancing clinical, functional, and/or personal recovery. The potential inter-relationships between these types of recovery are increasingly recognized, especially when treatment and support are linked with personal goals (2, 15). With few exceptions (18), most research concerning this consumer subgroup has evaluated individual service components, rather than the whole mental health system. Furthermore, there is a need for systematic reviews of the evidence for improving individuals' clinical, functional, and personal recovery through specific models of care. Using a whole system perspective, such a review should encompass models of care across both clinical and NGO sectors, as well as blended models of care [e.g., (19, 20)].

The term “model of care” (MoC), while not universally defined, broadly, describes the multi-dimensional way (21) health services are delivered (22). Ideally, a model of care should have a delivery component, defining how care is provided (including structural components of the model: hours of operation and staffing profile), and a content component, defining the treatment, care, and support that are delivered (23). Furthermore, an MoC should have a reasonable prospect of being able to offer much of the treatment, care, and support consumers might need, though it might be supplemented by other MoCs or interventions at times. Both inpatient and community-based MoCs have a role in achieving clinical, functional, and personal recovery outcomes. However, this review focuses on community-based models of care because of the increased emphasis on community-based care, with greater potential to support clinical, functional, and personal recovery, whereas inpatient units generally focus on the former.

This systematic review aimed to generate a narrative synthesis of recent evidence, regarding community-based models of care that support the clinical, functional, and personal recovery outcomes of people living with persistent and complex mental health needs. It contains varied language to describe the experience of severe and/or persistent mental illness or mental distress, including, for example, “patient,” “consumer,” “recovery,” and “rehabilitation.” The use of any term does not imply author endorsement but reflects the context of the studies and the extent of the review. This language and construction of the research issue may rightly be contested by consumers and their families; indeed, a limitation of a systematic review is only drawing from peer-reviewed literature which is, until now, almost entirely dominated by clinical and research constructions. We aimed to identify these challenges and alternative perspectives in this article.

## Methods

We conducted a systematic review of recent literature on models of care and interventions for individuals with severe and persistent mental illness and complex needs, for the Royal Commission into Victoria's Mental Health System in Australia (RCVMHS) (24). Elsewhere, we have provided a systematic review of a subset of identified studies reporting on the effectiveness of community-based models of care and interventions in supporting social inclusion for people living with severe mental illness (10), including supported accommodation, supported education, and supported employment. The present review reports on the effectiveness of community-based models of care, for individuals who experience persistent and complex mental health needs in supporting personal, functional, and clinical recovery outcomes. We did not preregister this review.

### Review team

The review team is deliberately interdisciplinary and includes researchers who bring their lived and living experiences of mental health issues and psychological distress. We learned from each other and evolved our understandings of different perspectives on important foundational concepts, in undertaking literature reviews. This includes what we consider to be evidence, its critical appraisal, and choice of language.

### Search strategy, and inclusion and exclusion criteria

Our search was conducted in October 2021 using Medline, EMBASE, PsycInfo, CINAHL, and Cochrane databases and included peer-reviewed articles published between October 2016 and October 2021. Our search terms (key words and MeSH terms) reflected three central concepts: “severe mental illness,” “models of care and/or interventions,” and “outcome and experience measurement” (full search string available upon request). These terms encompassed difficulties in day-to-day social and occupational functioning, co-existing conditions such as substance use and poor physical health, and other social experiences, such as unemployment and homelessness, which are likely to reflect persistent and complex mental health needs. We limited the search to publications in English and available in full text. Authors were contacted for relevant articles if the full text could not be accessed.

Inclusion criteria for the original RCVMHS search were (a) *models of care* (MoC) for adults aged 18–65 years *with severe and persistent mental illness*; and (b) *group or individual interventions* delivered alone or through an identified MoC. For example, Assertive Community Treatment (ACT) is a MoC (a form of intensive case management), whereas family psychoeducation is an intervention. Additional inclusion criteria for the present review were (c) community-based models of care with *a delivery component* that defines how care is provided and a *content component* that defines what treatment and care are delivered, in line with our previous definition (23); and (d) studies that evaluated MoCs *for people with severe mental illness* (SMI), defined as a primary diagnosis of schizophrenia, schizoaffective disorder, bipolar disorder, or other severe and enduring psychotic disorder.

Given the inconsistent terms to describe models of care, we adopted a broad definition for MoC. We used terms such as delivery of healthcare, continuity of patient care, quality of healthcare, model of care, and service delivery model. Since we aimed to identify MoCs in community settings, we included examples of integrated/coordinated care and transition pathways between hospital and community. *Community-based residential services* focused on clinical, functional, and/or personal recovery were also in scope. This included transitional residential rehabilitation MoCs if they were not registered as an inpatient ward. We also included studies of interventions (e.g., peer support or physical health interventions) but only when the intervention was described as augmenting or *enhancing a recognized and well-described MoC* (e.g., case management).

The individual-level outcomes of interest were clinical recovery (improvements in symptoms, insight, etc.); personal recovery [improvements related to illness self-management, discrimination, wellbeing, quality of life, and relational recovery (25)]; and functional recovery. We excluded service-level outcomes such as inpatient admissions and bed days as these were not capturing personal, functional, and clinical recovery outcomes *per se*. Similarly, we excluded days in stable housing and the number of days employed, respectively, for *supported accommodation* and *vocational support* MoCs. For MoCs other than *supported employment* or *supported accommodation*, we included employment or housing outcomes where they were used to assess functional recovery outcomes.

### Study selection

The results of the original search undertaken for the RCVMHS, updated in October 2021 to extend the study period to 5 years, were screened using the Covidence online software (https://www.covidence.org). After duplicates were removed, reviewers screened by title, abstract, and full text, with each study requiring two “yes” votes at the abstract and full-text screening stage to be included. Conflicting votes were resolved through consulting with the project leads (CH and LB).

### Quality of evidence

The included articles were evaluated by the Kmet standard criteria to assess the methodological quality of both quantitative and qualitative research (26). Quantitative papers were rated on 14 items and qualitative papers on 10 items, related to the study design; participant selection; data analysis methods; and the clarity and interpretation of results. Each article was rated by one reviewer and validated through discussion between reviewers at regular meetings, to ensure consistency in rating. Total scores were reported out of 100 (i.e., as percentage equivalents), to take account of non-applicable items.

We developed a data extraction table and guidance notes, to enhance consistency in the description and synthesis of findings from studies. Studies were grouped according to MoCs derived from our original RCVMHS review and collective knowledge of this area of service delivery, and each co-author produced a textual summary for one model. To ensure consistency, the textual summaries were refined and finalized through consensus discussion across the author group. A descriptive overview of the features and findings for each MoC was then generated. This allowed for further scrutiny and reorganization of study groupings and their optimum placement within the final set of MoCs.

### Narrative synthesis

Given the range of MoCs included, we chose a narrative synthesis as the most suitable approach to summarize our findings. Narrative synthesis includes a preliminary synthesis to identify patterns of findings across studies; exploration of whether effects of an intervention vary according to study population; identification of factors influencing the results within individual studies and explaining differences between studies; development of a theoretical framework underpinning specific intervention effects; assessment of the robustness of the synthesis based on the strength of evidence; and discussion of the generalizability of conclusions to wider populations and contexts (27). Each model was rated and categorized as either best practice, promising practice, or emerging practice, according to an overall rating of the level of evidence (28). Since our review included multiple MoCs, we did not aim to develop a theoretical framework underpinning the effects of each model. Our four researchers with lived experience led the narrative synthesis.

## Results

**Figure 1:**
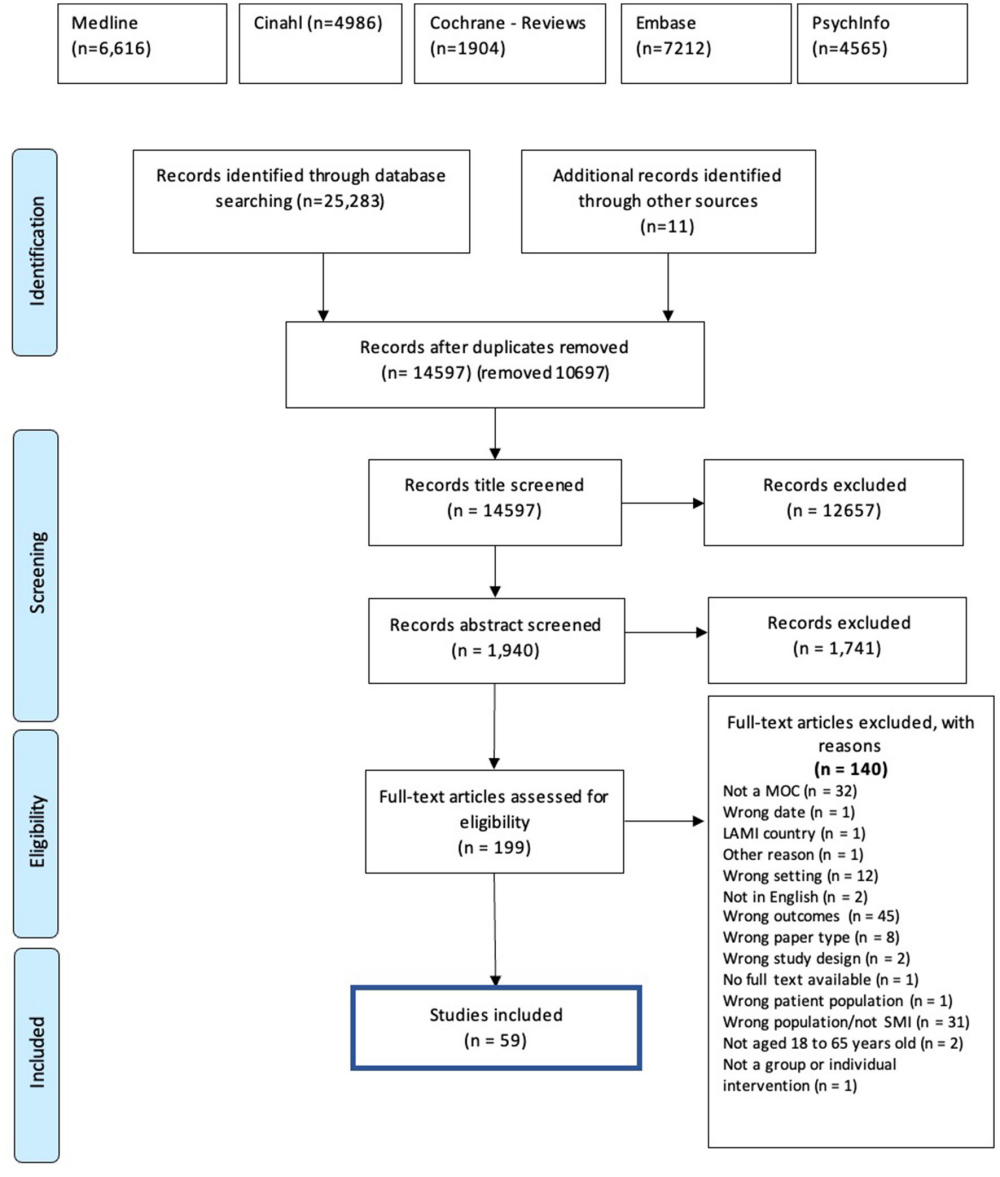
PRISMA flow chart.

Fifty-nine studies that met our inclusion criteria (46 quantitative,10 qualitative, three mixed methods; PRISMA flowchart in Figure 1; summary of study characteristics in Table 1) across 20 high-income countries are Australia (11), Brazil (1), Canada (4), Chile (1), Denmark (3), France (1), Germany (1), Hungary (1), Ireland (1), Israel (2), Italy (1), Netherlands (8), Norway (1), Poland (1), Spain (2), Switzerland (1), Taiwan (1), Turkey (2), UK (2), and US (14). Sample sizes ranged from 15 to 4,216 participants. The average Kmet score for quantitative papers was 68 (range = 10–95) and for qualitative papers was 76 (range = 40–100) (Tables 2A, B).

**Table 1:** Models of care clusters, definitions, and how recovery outcomes were measured.

**Type of model of care [Country (citations) (no)]**	**Definition of models of care or programs included**	**Recovery outcomes (and how measured)**		**Key qualitative findings**	**Summary of recovery-related findings**
		**Personal recovery outcomes**	**Other outcomes (clinical and functional)**		
**Goal-focused (*****n*** **=** **4)** Brazil (29) (1) Denmark (30) (1) Ireland (31) (1) USA (32) (1)	Interventions primarily focused on goal identification and pursuit. Includes one self-directed budget model, and one Internet-delivered program.	Personal recovery (RAS, Bipolar Recovery Questionnaire, BRQ), quality of life (Quality of Life in Bipolar Scale, QOL.BD), self-esteem (Empowerment Scale), control over one's life (CMS), autonomy (PASS, ILSS answered by caregivers)	Symptoms (BSI, PANSS), severity of symptoms (Internal State Scale, ISS), insight (BIPQ), occupation (employment and education), social and functional behavior (DAFS-R)	Illness Management and Recovery (IMR) program participants noticed small impacts in everyday life and broke barriers to goal attainment, but the high structure did not suit everyone—some needed additional flexibility or personal tailoring allowing them to pause, repeat, or postpone.	The identified quantitative studies (RCT and pre-post designs) suggested that focused interventions within ongoing care models impact the target, e.g., executive function (29) or treatment self-management (31)—but these changes may not impact recovery. A specific focus on the person's own goals was associated with positive recovery (33), as was greater self-direction of recovery support resources (32). Qualitative data suggested that contextual factors and non-linear experiences could derail the pursuit of goals and impede recovery (31, 33), even within a well-defined, goal-pursuing intervention such as IMR (33). Flexibility and ample timeframes are required for effective goal-setting to support recovery (32, 33). One RCT (29) lacked personal recovery measures or any consumer perspectives.
**Integrated community treatment (including case management) (*****n*** **=** **10)** Australia (34) (1) Germany (35) (1) Israel (36) (1) Spain (37) (1) Taiwan (38) (1) Turkey (119] (1) USA (39–42) (4)	Community-based case management typically incorporates assessment of need, extended pharmacological and social interventions, and monitoring, integrated with a range of adjunctive interventions that may include—peer support, physical healthcare, skills training, or cognitive remediation. Some models could flex intensity according to need but to a lower intensity than ICM.	Personal recovery (RAS), self-efficacy (GSES), empowerment (Empowerment Scale, RA), connectedness (Sense of Community Index), wellbeing and QoL (WHO-5, QoL BD, MANSA, QLS, ANSA, MSQL, EUROHIS-QOL), and hope (HS)	Symptoms (PANSS, CGI, HDRS, HAM-A, YMRS, CSI, PHQ-9, NOMS, BPRS), functioning (GAF, FAST, FROGS, ANSA, SF-36, NOMS), social functioning (Modified Social Functioning Scale, SCI), and insight (SAI)	Integrated models of health, mental health, and social care are highly acceptable to consumers/users, with the potential to enhance durations of engagement.	Strength-based case management (36) and case management enhanced with peer support (39) demonstrated positive outcomes for personal recovery, including quality of life. There were significant improvements reported in functional (37, 38, 40) and clinical (37, 40) recovery for case management approaches with integrated enhancements. Integrated models of health, mental health, and social care are highly acceptable to consumers/users, with the potential to enhance durations of engagement (39). Higher engagement showed positive impacts on clinical and functional recovery (43).
**Intensive case management (*****n*** **=** **11)** Australia (44) (1) Canada (45) (1) Netherlands (46–50) (5) Norway (51) (1) Switzerland (52) (1) Turkey (53) (1) US (54) (1)	Including assertive community treatment (ACT), offering daily contact in the person's home and support outside office hours to help them avoid hospital admission and assist with various aspects of their ongoing recovery, and Flexible ACT (FACT)—lower intensity support with the capacity to increase intensity as needed. Also looked at technological enhancements to ACT or FACT. One study of intensive home treatment for people in crisis.	Personal recovery (RAS) non-standardized measure of goal attainment, experienced discrimination (Discrimination and Stigma Scale), self-efficacy, recovery and empowerment (MHCS), victimization (Dutch Safety Monitor), quality of life (MANSA, EQ-5D, WHOQoL-brief), carer burden (Zarit Caregiver Burden Scale)	Housing stability, meaningful occupation and functioning (HoNOS, GAF), illness severity (CGI), symptoms (HoNOS, BDI, BAI, PSYRATS, GPTS, BPRS, SAPS, SANS), illness management (IMRS), employment, functioning (GAF, PSF), suicidality, social functioning (SFAS, MCAS), social participation (SFS)	NA	Ongoing interest in ICM for those with more severe problems was evident from the relatively high number of identified studies evaluating ACT and FACT, alongside one study from a less economically developed country evaluating an enhanced form of community mental health care for this group (53). Several of these studies reported significant clinical, functional, and personal recovery outcomes (50, 52, 53). Two studies reported that the gains made through ACT were sustained after discharge to other community services (45, 52). Another study suggested ACT was as effective for people with substance misuse as those without (51). An uncontrolled retrospective review of agency records suggested ACT supported young adults to engage with employment and education (54). However, while there is growing evidence for FACT (48, 50), including enhancements to support recovery and minimize discrimination (48), there remains a lack of definitive RCTs. Feasibility studies suggest that ACT and FACT could be augmented with digital technology (47, 49).
**Partners in recovery care coordination (*****n*** **=** **3)** Australia (55–57) (3)	Programs focused on driving collaboration, coordination, and integration between services and support from multiple sectors. Describing a particular model funded in Australia from 2011 to 2019 called Partners in Recovery that targeted people with severe and persistent mental illness and complex needs and their carers and families.	Personal recovery (RAS-DS, Canadian Institutes of Health Information Measuring Patient Experiences in Primary Health Care Survey)		Increased access to coordination, increased participation in education, employment, and housing stability, as well as more involvement in treatment decisions, lead to consumers feeling more hope and self-efficacy. Support facilitators central to enhanced coordination.	Partners in Recovery demonstrated positive outcomes for personal recovery (55) housing stability and increased participation in employment and education (56). High involvement in recovery planning resulted in increased self-efficacy, reduced experience of stigmatization (57), and greater confidence in managing their illness (56, 57).
**Rehabilitation and Recovery-focused (*****n*** **=** **8)** Australia (58–61) (4) Italy (62) (1) Netherlands (63) (1) Poland (64) (1) USA (65) (1)	Rehabilitation and recovery-focused programs of varying lengths and settings, including a residential farm, Transitional Residential Rehabilitation (TRR) and sub-acute residential programs, and enhancements to rehabilitation programs.	Self-efficacy (GSES), quality of life (MANSA, AQol-8D, Lehman QLS), and personal recovery (RAS)	Symptoms (BASIS-24/32, BPRS, CGI, HoNOS, and PANSS), social functioning/problems (HoNOS, SFS), functioning (GAF, BASIS-24/32) relational and independent living skills (Lehman QLS), paid work, life skills (LSP-16), social participation (SFS, Dutch National Societal Participation Ladder), insight (BCIS, AIS), and accommodation instability	Being in an inclusive recovery community is valued, with both other residents and staff contributing to the sense of community. The environment can support activity engagement, with the recognition that doing things is important and supports recovery.	The identified quantitative studies, mostly uncontrolled pre-post designs (60–62, 65), suggested that these models hold promise for clinical and functional recovery, with significant findings in five of six studies (60–62, 64, 65). Neurofeedback was more effective in improving consumers' self-efficacy when added to standard rehabilitation, according to one RCT (64). Social participation improved in the other RCT (63) but to an equivalent extent in the rehabilitation and active control conditions. Except for the latter study, personal recovery measures were absent from quantitative studies, but two qualitative studies emphasized the importance of fostering inclusive communities (58) and supporting meaningful activity to support personal recovery within TRR models of care (58, 59).
**Social and community connection-focused (*****n*** **=** **8)** Canada (66) (1) Chile (67) (1) Hungary (68) (1) UK (33) (1) US (69–72) (4)	Interventions with a clear focus on social or community connectedness outcomes. Five studies had a social mode of delivery [intervention was primarily delivered in a social context such as Clubhouse (4) or multi-family group (1)], while other interventions included peer support (3) directed at strengthening social connection/group involvement.	Personal recovery (RAS, Personal recovery outcome measure), empowerment (The Empowerment Scale), quality of life (SLS and LQoLI), self-esteem (RSES), identification, and pursuit of goals	Illness management, symptoms (PANSS, BSI, and CSI), social (ESIS and SPS) and functional (DRFS-R, IMR, and SDS-3) behaviors, community functioning/integration (Multnomah Community Ability Scale, Community Integration Scale), social support (Social Support Survey), global functioning (GAF), community participation (TUCPM), independent living (carer report), perceived familial support (FAPS), social confidence, and vocational outcomes	Culturally relevant concepts can ensure the relevance of interventions for specific communities (e.g., Pilinaha framework in Hawaii), although culturally adapted elements of the family group intervention were not detected by most participants (33). People use Clubhouse for connection, belonging, skill building, and daily structure—they attend voluntarily because they see wellbeing as inherently social. Community/in-home support is generally valued, although stigma impacted the comfort of having support at home for some. Combined peer and professional support was valued. Some programs are too brief.	Socially delivered interventions, such as Clubhouse, are valued by participants as supporting personal recovery (70, 71). People were motivated to attend because they associate having structure, connections, and building skills with contributing to wellness and recovery (71). One RCT comparing Clubhouse and TAU (case management) showed functional improvements in both groups (68). Small sample sizes and low engagement may have contributed to the null findings for functional and personal recovery of individually delivered peer supports (66, 69). Peer and professional in-home support was generally feasible and acceptable in Chile, although stigma decreased the comfort of home-based contact for some (67). Culturally relevant concepts can ensure the relevance of interventions for specific communities (33, 67, 70).
**Supported accommodation (*****n*** **=** **7)** Australia (73) (1) Canada (74, 75) (2) France (76) (1) Netherlands (77, 78) (2) USA (79) (1)	Enhanced housing support—including sheltered housing, housing with family, living alone in social housing, or private rental. One approach tested enhanced staff training with a comprehensive approach to rehabilitation (CARe) and one involved the addition of peer support.	Victimization prevalence (crime victimization scale of the DCV), quality of life (HoNoS and MANSA), wellbeing (Lehman's 20-item QOL interview), personal recovery (MHRM and RAS-22), empowerment (DES), hope (HI), and self-efficacy (MHCS)	Symptoms (CSI), social functioning (HONOS social subscale, SFS), clinical recovery and overall functioning (HONOS), community functioning (MCAS), and community integration (CIS)	Stable housing is “a refuge” that prompts reflection on the past and hopes for the future. Peer specialist and non-peer specialist providers can assist in enhancing healthy lifestyles. Differences were identified in approach to practice, power dynamics, and shared experience vs. shared goals. Peer specialists were less biomedical and encouraged the free expression of emotion and hopefulness.	The quantitative studies (73–75, 77, 78), including two RCTs (75, 77), demonstrated mixed results in relation to outcomes other than housing stability, which were often secondary outcomes. Victimization prevalence was highest among residents in sheltered housing compared to people living alone or with family (78). Dunt et al. (73) found significant positive outcomes on the overall HoNOS score and the social subscale. In two follow-up studies: women and non-white participants achieved better wellbeing trajectories over 6 years (74); and, those in congregate Housing First (HF) as opposed to scattered-site HF or TAU experienced more significant improvement in community integration and recovery after 24 months (75). The effects of HF are considerable (76) but they are not always enough to prevent a negative trajectory. While peer specialists supported recovery-related outcomes (79), training staff in a comprehensive approach to rehabilitation (CARe) did not lead to more improvement in clients' personal recovery and social functioning (77).
**Vocational support (*****n*** **=** **8)** Australia (80) (1) Denmark (81, 82) (2) Israel (83) (1) Spain (84) (1) UK (85) (1) USA (86, 87) (2)	Individual placement and support (IPS), individual placement support with enhancements (IPSE), IPS with work-focused CBT, and cognitive remediation and enhanced vocational rehabilitation.	Self-efficacy (GSES), self-esteem (Rosenberg SES), quality of life (MANSA, QoLI, AQoL-6D, and EQ-5D), wellbeing (WEMWBS), personal recovery (QPR), perception of self as a worker, and self-confidence	Symptom severity (SANS, SAPS, SF-12, PANSS, and HAM-D), social functioning (Personal and Social Performance Scale, Social Skills Performance Assessment), functioning, insight (IMR), living skills (ILSS), and effects of symptoms on daily functioning (SDS-3)	IPS and competitive work might have an impact on personal recovery, and decrease negative and depressive symptoms, but does not seem to have an impact on psychotic symptoms.	Most of the quantitative studies investigated the effects of IPS or another vocational service enhanced with cognitive remediation (CR) and/or social skills training and/or CBT (80, 81, 84–87). Three of the RCTs (81, 84, 85) showed no additional benefits for these enhancements, whether for personal, functional, or clinical recovery. A trial of compensatory cognitive training in the context of supported employment resulted in initial improvements in depressive symptoms and quality of life which were not sustained (87). Improved wellbeing and quality of life (80) and marginal clinical improvements (86) were reported in pre-post studies, both testing forms of CR in vocational services. Consumers reported they experienced better functioning in supported employment as opposed to vocational centers and sheltered workshops (83). The sole qualitative study (82) suggested that IPS and competitive work have a positive impact on personal recovery and depressive symptoms.

We excluded studies that were (a) conducted in environments other than the community (e.g., prisons or hospitals); (b) studies that focused on individuals without a diagnosis of SMI (e.g., personality and mood disorders, substance use disorder, acquired brain injury, intellectual disability, or trauma); (c) studies where fewer than 50% of the sample met our SMI diagnostic inclusion criteria (see above); (d) studies that did not report on any relevant individual recovery outcomes, whether clinical, functional, or personal; (e) studies conducted in low- and middle-income countries; (f) publications that did not report primary empirical data; and (g) gray literature.

**Table 2A:** Quantitative Kmet scores by paper group.

**Paper group**	**References**	**Kmet %**
**Rehabilitation and recovery-focused**		
	Heatherington et al. (65)	86
	Markiewicz et al. (64)	69
	Nibbio et al. (62)	73
	Parker et al. (60)	82
	Sanches et al. (63)	82
	Thomas et al. (61)	68
**Intensive case management**		
	Albers et al. (48)	95
	Barakat et al. (46)	70
	Ben-Zeev et al. (47)	80
	Blankers et al. (49)	70
	Clausen et al. (51)	70
	Incedere et al. (53)	35
	Iyer et al. (45)	80
	Klodnick et al. (54)	10
	Nugter et al. (50)	65
	Siskind et al. (44)	55
	Vidal et al. (52)	45
**Integrated community tr eatment (including case**		
**management)**		
	Corrigan et al. (39)	60
	Errichetti et al. (41)	70
	Gelkopf et al. (36)	80
	Li et al. (38)	40
	Mahlke et al. (35)	85
	Nibbio et al. (62)	73
	O'Connell et al. (42)	75
	Palmer et al. (34)	95
	Sahin et al. (43)	50
	Soberay et al. (40)	50
	Valls et al. (37)	45
**Partners in recovery care coordination**		
	Banfield et al. (57)	50
	Hancock et al. (55)	65
**Goal-focused**		
	Cook et al. (32)	65
	Enrique et al. (31)	55
	Vizzotto et al. (29)	58
**Supported accommodation**		
	Bitter et al. (77)	80
	Dunt et al. (73)	70
	Mejia-Lancheros et al. (74)	70
	Somers et al. (75)	80
	Zarchev et al. (78)	60
**Vocational support**		
	Christensen et al. (81)	75
	Gal et al. (83)	86
	McGurk et al. (86)	70
	Miles et al. (80)	55
	Rodriguez-Pulido et al. (84)	90
	Schneider et al. (85)	70
	Twamley et al. (87)	75
**Social and community connection-focused**		
	Agner et al. (70)	90
	Kidd et al. (66)	90
	Gumber et al. (72)	61
	Salzer et al. (69)	60
	Varga et al. (68)	92

**Table 2B:** Qualitative Kmet scores by paper group.

**Paper group**	**References**	**Kmet %**
**Rehabilitation and recovery-focused**		
	Parker et al. (58)	80
	Rees et al. (59)	80
**Intensive case management**		
	NA	NA
**Integrated community treatment (including case**		
**management)**		
	NA	NA
**Partners in recovery care coordination**		
	Banfield et al. (57)	70
	Isaacs et al. (56)	75
**Goal-focused**		
	Enrique et al. (31)	80
	Jensen et al. (33) exp of goal-setting	65
**Supported accommodation**		
	Bochicchio et al. (79)	90
	Rhenter et al. (76)	100
**Vocational support**		
	Gammelgaard et al. (82)	80
**Social and community connection-focused**		
	Agrest et al. (67)	80
	Jensen et al. (33)	40
	Pernice et al. (71)	90
	Salzer et al. (69)	60

The studies considered a range of clinical, functional, and personal recovery outcomes. More measurement efforts focused on clinical recovery—symptoms, psychological distress, and illness severity—and functional recovery—general functioning, employment, housing stability, and social functioning. Personal recovery was assessed by a range of measures, some of which had clear evidence of co-design with people with lived and living experience, whereas others, such as the General Self-Efficacy Scale (88), did not. Qualitative studies noted factors relevant to personal recovery including hope, empowerment, connectedness, and inclusion. Where social outcomes were measured, this was more typically through social skills/functioning (functional recovery) than through the establishment of meaningful social connections (personal recovery).

Methods of assessing outcomes varied considerably (see Table 1) and included both standardized measures, subscales or individual items from standardized measures, or uniquely developed measures for individual studies. The specificity or generality of outcomes varied considerably across studies, making comparison and integration of findings challenging. For example, measurement of functioning varied from global (e.g., Global Assessment of Functioning, GAF) (89) to fine-grained (e.g., social functioning using the Social Functioning Assessment Scale, SFAS, the External Social Integration Scale, ESIS, and the Social Provisions Scale, SPS) (90–92).

### Model of care characteristics and study findings

Papers evaluating different MoCs were grouped as follows: *goal-focused*; *integrated community treatment (including case management)*; *intensive case management*; *partners in recovery care coordination*; *rehabilitation and recovery-focused*; *social and community connection-focused*; *supported accommodation*; and *vocational support*. Each of these is defined in Table 1, along with a high-level summary of the findings per group. Details of each study's findings and corresponding details of the delivery and content characteristics of the researched MoC are presented in Tables 3, 4, respectively. Most MoCs either had some examples of team delivery (e.g., *integrated community treatment*) or were explicitly a team-based approach (e.g., *partners in recovery care coordination*; see Table 4). Where studies included a comparison to treatment as usual (TAU), this was often poorly described [e.g., (37, 45, 46)].

**Table 3:** Details of the individual studies within the included models of care.

**References**	**Country**	**Study setting**	**Aims/ hypotheses/ research questions**	**Paradigm (quantitative/ qualitative/ mixed methods)**	**Study design**	**Participants *N* (intervention = *n* and control = *n*)**	**Mean age (SD) gender male %**	**Outcomes**	**Key findings**
**Goal-focused**									
Cook et al. (32)	USA	Community	Examine effects of self-directed care financing on outcomes, service costs, and user satisfaction among adults with serious mental illness	Quantitative	RCT	*N* = 217 (intervention = 115 and control = 102)	41.6 (9.7) 38%	Primary: Personal recovery (RAS). Secondary: Self-esteem (SES), control over one's life (CMS), autonomy (PASS), and psychiatric and somatic symptoms (BSIGSI).	Intervention participants reported significantly improved personal recovery, self-esteem, coping mastery, autonomy support, somatic symptoms, employment, and education compared to TAU.
Enrique et al. (31)	Ireland	Two secondary-care services, community	Examine the feasibility, acceptability, and preliminary efficacy of implementing an Internet-delivered, clinician-supported intervention for bipolar disorder as an adjunct to TAU	Mixed methods	Uncontrolled pre-post	*N* = 15	40.2 (12.0) 77%	Personal recovery [the bipolar recovery questionnaire (BRQ)], quality of life (QOLBD), severity of symptoms (ISS), insight (BIPQ), early warning signs (bespoke), and semi-structured interviews with participants explored changes since starting the program.	There was a significant improvement in patients' sense of personal recovery. A relatively small number of completed participants found the program helpful. No statistically significant findings on QoL, severity of symptoms, or early warning signs.
Jensen et al. (33)	UK	NHS inpatient or community care	Determine the acceptability of a Culturally Adapted Family Intervention (CaFI) as an appropriate and beneficial intervention for African-Caribbean service users with psychosis and their families (or proxy family members—volunteers selected by person)	Qualitative	Semi-structured interviews	*N* = 34 (service users = 22 and family = 12)	Not reported Not reported	Perception of need and benefits of CaFI.	Service users reported experiencing personal and interpersonal benefits, such as an increase in confidence in social settings and learning adaptive coping mechanisms. CaFI is viewed as informative, educational, and effective at normalizing symptoms. Improvement in family dynamics, coping skills, and knowledge of diagnosis.
Vizzotto et al. (29)	Brazil	Outpatient service	Test the hypothesis that the Occupational Goal Intervention method effectively improves executive functioning in people with treatment-resistant schizophrenia (TRS)	Quantitative	Single-blind RCT	*N* = 48 (intervention = 26 and control = 22)	38.0 (8.5) 79%	Primary: Social and functional behaviors (DAFS-R). Secondary: autonomy in activities of daily living (ADLs) (ILSS answered by caregivers) and clinical recovery (PANSS).	Improved social and functional behaviors were found in people with TRS. The greatest effects were in the families' and caregivers' perceptions of participants' autonomy and living skills in regard to ADLs. The effect size for clinical recovery was small.
**Integrated community treatment (including case management)**									
Corrigan et al. (39)	USA	Community-based mental health services	Measure the impact of a peer navigator program on the health needs of Latinos with serious mental illness	Quantitative	RCT	*N* = 110 (intervention = 55 and control = 55)	Intervention = 48.6 (9.9) and control = 42.7 (11.9) Intervention = 48%, control = 33%	Recovery (RAS), empowerment (RA), quality of life (QLS).	Improvement in quality of life and recovery scales but not empowerment scale.
Errichetti et al. (41)	USA	Community	Examine the effects of reverse collocated integrated care on persons with SPMI and comorbid chronic disease receiving behavioral health services at a local mental health authority	Quantitative	RCT	*N* = 426 (intervention = 249 and control = 167)	Not reported Not reported	Secondary: Depression (PHQ-9), functioning (ANSA), and QoL (ANSA).	The intervention was not found to significantly impact depressive symptoms. No follow-up data on the ANSA were reported.
Gelkopf et al. (36)	Israel	Community	Assess the effectiveness of a new strength-based case management (SBCM) service in Israel, using a randomized controlled approach	Quantitative	RCT	*N* = 1,276 (intervention = 696 and comparator = 580)	39.2 60%	Quality of life (adaptation of MANSA), interpersonal relationships (adaptation of MANSA), self-efficacy (bespoke), goal-setting and attainment (bespoke), and psychiatric symptoms (CSI).	SBCM participants improved in self-efficacy, and general quality of life, and set more goals than the control group. No difference in psychiatric symptoms. Psychiatric Rehabilitation Services: participants' satisfaction with their interpersonal relationships decreased significantly than TAU.
Li et al. (38)	Taiwan	Community homecare	Integrate effective evidence-based community care services that are subjected to heavy caseloads and then to examine the effects on individuals with schizophrenia	Quantitative	Test–retest experimental design	*N* = 85 (intervention = 50 and TAU = 40)	45.8 57%	Functioning (GAF).	Significant improvements in general functioning levels in the intervention, compared to the comparison group.
Mahlke et al. (35)	Germany	Interventions carried across community mental health and inpatient settings	Test the effectiveness of one-to-one peer support (with defined roles and extensive training for peer supporters), compared with TAU, regarding self-efficacy	Quantitative	Pre-post	*N* = 216 (intervention = 114 and control = 102)	41.5 43%	Self-efficacy (German language version of the General Self-Efficacy Scale) and QoL (MSQL).	Self-efficacy was significantly greater for the intervention group. QoL improved for the intervention group.
O'Connell et al. (42)	USA	One public community mental health service.	Evaluate the addition of a peer mentorship program to standard care for people with SMI who were high users of inpatient care	Quantitative	RCT	*N* = 77 (intervention = 44 and control = 33)	40 51%	Social and community functioning (MSFS and SCI), psychiatric symptoms (BPRS), functional health and wellbeing (SF-36), hope (HS), and sense of community (SCI).	No significant main effects or interactions were observed between the intention-to-treat and control groups in functional health, hope, or sense of community. Participants who attended >1 Recovery Mentor session improved in social and community functioning. Intent-to-treat and treated samples showed significant improvements in psychiatric symptoms.
Palmer et al. (34)	Australia	Community	Assess whether an adapted mental health experience co-design intervention to improve recovery orientation of services led to greater psychosocial recovery outcomes for service users	Quantitative	Stepped wedge cluster RCT	*N* = 468 (service users = 287, carers = 61 and staff = 120)	37 63%	Primary: Individual psychosocial recovery (RAS-R); Secondary outcomes: QoL (EUROHIS-QoL), qualitative data including health timeline, a week-in-the-life diary, and a social network map.	No difference between intervention and controls: RAS-R scores were similar between the intervention and control phases. Qualitative evaluation feedback gathered from all post-group meetings suggested that participants found value in feeling heard, being involved in decisions about service improvements, and working together.
Sahin et al. (43)	Turkey	Community	Determine the effects of participation frequency in a CMHC on insight, treatment adherence, and functionality in severe mental disorders	Quantitative	Retrospective descriptive	*N* = 362	41.5 (12.7) 62%	Clinical recovery (CGI), functional outcomes (GAF, FROGS), and insight (SAI).	There was a significant difference between the baseline and the end of 1 year in all assessments. Increasing CMHC participation was associated with improved insight and functioning in those living with psychosis and those with bipolar disorder at the end of 1 year.
Soberay et al. (40)	USA	Community	Evaluation of physical and social health outcomes for users of an integrated care clinic	Quantitative	Pre-post program evaluation over 4 years	*N* = 534	Not reported 39%	Everyday functioning (NOMS), psychological distress (NOMS), and social connectedness (NOMS).	Statistically significant improvement over time in everyday functioning, psychological distress, and social connectedness.
Valls et al. (37)	Spain	Outpatient mental health unit	Test the efficacy of a novel adjunctive treatment in patients with bipolar disorder, including psychoeducation, mindfulness training, and functional remediation	Quantitative	RCT	*N* = 94 (intervention = 47 and control = 47)	Intervention = 47.6 (7.3), TAU = 45.8 (9.9) Intervention = 57%, control = 41%	Primary: Psychosocial functioning (FAST). Secondary: Depressive symptoms (HDRS), anxiety symptoms (HAM-A), manic symptoms (YMRS), wellbeing and quality of life (WHO-5–Spanish), quality of life (QoL.BD–Spanish).	Significant improvements in two of the six domains of psychosocial functioning and depressive symptoms in the intervention group, compared to TAU. No significant findings for other outcomes of interest.
**Intensive case management**									
Albers et al. (48)	Netherlands	FACT team office or patients' homes	Assess the effectiveness of a new intervention to manage and prevent revictimization, and support safe social participation	Quantitative	Cluster RCT	*N* = 400 (intervention = 216, control = 184)	Intervention = 44.4 (9.5), control = 46.6 (10.0) Intervention = 62%, control = 60%	Primary: Social participation (SFS), victimization (Dutch Safety Monitor), and discrimination (Discrimination and Stigma Scale). Secondary: Acknowledgment of difficulties and support in recovery, self-efficacy, and empowerment (Mental Health Confidence Scale), quality of life (MANSA), and psychosocial functioning (HoNOS).	No significant differences in social functioning. Experienced and anticipated discrimination and self-efficacy increased slightly in both groups. There were small but significant positive time-by-condition interactions after 20 months for experienced discrimination and acknowledgment of difficulties and support in recovery in the intervention group. No significant differences were found for other outcome measures.
Barakat et al. (46)	Netherlands	Community, home-based	Test whether providing intensive home treatment to patients experiencing a psychiatric crisis results in a stronger increase in self-efficacy when compared to TAU	Quantitative	RCT	*N* = 142 (intervention = 93, control = 49)	41.5 (12.0) 43%	Self-efficacy (Mental Health Confidence Scale).	No difference in self-efficacy between Intensive Home Treatment and TAU at 26 weeks.
Ben-Zeev et al. (47)	Netherlands	Online	Evaluate the feasibility and clinical utility of training intensive psychiatric community care team members to serve as “mobile interventionists” who engage patients in recovery-oriented texting exchanges	Quantitative	Pilot RCT	*N* = 49 (intervention = 37, control = 12)	44.8 (11.2) 55%	Feasibility, acceptability, clinical outcomes (BDI, BAI, PSYRATS, and GPTS), illness management (IMRS), and recovery (RAS).	Use of online tools is feasible and acceptable. At 3 months post-intervention, better symptom management, clinical outcomes, and recovery in the intervention group. Significant difference in medium effect size for BDI and GPTS. Small significant effect sizes IMSR and RAS. Between-group effects were non-significant. The 6-month follow-up revealed that gains were not maintained after the intervention was discontinued.
Blankers et al. (49)	Netherlands	Community FACT	Compare treatment satisfaction, clinical outcome, and quality of life in the short term of patients receiving blended (combined face-to-face and Internet-based) FACT with those receiving conventional FACT	Quantitative	Open-label prospective controlled cohort study	*N* = 47 (intervention = 25, control = 22)	Intervention = 44.4 (9.5), control = 48.9 (10.2) Intervention = 44%, control = 50%	Clinical outcomes (HONOS), quality of life (MANSA, EQ-5D), and self-efficacy beliefs (MHCS).	Blended FACT intervention leads to comparable improvements in quality of life and self-efficacy belief outcomes compared with standard FACT.
Clausen et al. (51)	Norway	Patient home and ACT office	Explore if outcomes associated with rehabilitation changed for patients both with and without problematic substance use after 2 years with ACT	Quantitative	Comparative cohort study	*N* = 142 (substance misuse = 84, no substance misuse = 58)	40 (8.7) 67%	Housing situation, occupation and activities, psychiatric symptoms (BPRS-E), functioning (GAF, PSF), and QoL (MANSA).	Housing, functioning, and anxiety and depressive symptoms improved in both groups at follow-up. There were no differences between groups in outcomes, except a reduction in manic symptoms in the substance use group.
Incedere et al. (53)	Turkey	Community, home-based, and other community settings	Conduct a case management model (hybrid of clinical CM, rehabilitation-oriented CM, and intensive case management) on a group of individuals with SMI and evaluate the outcomes during a 24-month follow-up	Quantitative	Service-level evaluation with an uncontrolled observational follow-up, comparing pre- and post-service change	*N* = 34	35.5 (8.7) 77%	Severity of illness (CGI-S), functioning (GAF), social functioning (SFAS), and caregiving (Zarit Caregiver Burden Scale).	All patients improved in clinical outcome and social functioning compared to pre-intervention. Family burden was decreased. Ten patients became employed and three patients left work.
Iyer et al. (45)	Canada	Community	Investigate whether individuals with first-episode psychosis receiving extended early intervention (EI) for 5 years were less likely to experience suicidal ideation and behaviors than those transferred to regular care after 2 years of EI	Quantitative	Secondary analysis of RCT data	*N* = 220 (intervention = 110, control = 110)	22.4 (4.4) 69%	Suicidality (BPRS), positive, negative, and depressive symptoms (SAPS and SANS).	No difference in suicidality between groups over the 5 years.
Klodnick et al. (54)	USA	Contacts usually made in clients' homes or elsewhere in the community	Describe participant characteristics; explore participant goal types, prevalence, and progress; and, track education and employment engagement and psychiatric hospitalizations	Quantitative	Uncontrolled service evaluation, using agency clinical records	*N* = 110	21.9 (2.2) 60%	Individual goal attainment and work and school engagement.	Independent of enrollment length (6, 12, or 24 months), participants on average made progress on 80% of their collective goals. Of those enrolled at least 6 months, 27% obtained employment or enrolled in school in the first 6 months of enrollment. Of those enrolled 24 months or more, 65% obtained employment or enrolled in school.
Nugter et al. (50)	Netherlands	Community—FACT	Investigate social and clinical outcomes and use of care during and after implementation of FACT	Quantitative	Cohort	*N* = 298	44.1 (12.2) 56%	Fidelity (FACTs), remission (remission tool based on PANSS), psychosocial functioning (HoNOS), quality of life (MANSA), and markers of social inclusion.	At follow-up, there was a statistically significant improvement in quality of life. An interaction between duration of ACT and improvement in symptoms. Social problems and quality of life were found.
Siskind et al. (44)	Australia	Community	Investigate factors associated with discharge from ACT and time with ACT	Quantitative	Retrospective review of electronic records	*N* = 167	32.5 (11.2) 59%	Symptoms (HoNOS) at discharge.	Symptoms decreased between entry and discharge.
Vidal et al. (52)	Switzerland	Community	Evaluate patients' long-term clinical and psychosocial evolution after discharge from ACT	Quantitative	Cohort	*N* = 29	48.8 (10.6) 55%	Symptoms (BPRS), quality of life (WHOQOL-BREF), social functioning (MCAS), and personal recovery (RAS).	Following discharge from ACT (mean 6.3 years), patients sustained improvement in their symptoms, quality of life, and social functioning achieved while with ACT.
**Partners in recovery care coordination**									
Banfield et al. (57)	Australia	Community	Evaluate the processes and outcomes of the Partners in Recovery (PIR) initiative in the Australian Capital Territory, a program established to improve the coordination of health and social care for this population	Mixed methods	Evaluation	*N* = 41 (clients *n* = 25, service providers *n* = 14 and carers *n* = 2)	42.8 (12.5) 28%	Recovery outcomes (including impacts of care in relation to the sense of control, feeling recovery plan would make a difference, and confidence in the ability to take care of self) (Canadian Institutes of Health Information Measuring Patient Experiences in Primary Health Care Survey and qualitative interviews).	At least 80% of clients reported positive impacts of PIR on recovery outcomes at the midpoint and endpoint of program utilization. There was a decrease in feeling that the recovery plan would make a difference and confidence in self-care from midpoint to endpoint, which may be related to uncertainty about the program's future. A major theme in interviews was the destigmatizing nature of the program, which helped people with their sense of self-efficacy.
Hancock et al. (55)	Australia	Community	Examine whether consumers engaged in PIR programs in two large regions of Sydney experienced a reduction in unmet needs (either via self- or staff report) and progress in their self-reported mental health recovery	Quantitative	Pre-post	*N* = 703	42.7 (11.1) 50%	Recovery (RAS-DS).	Consumers experienced positive changes in their recovery during their engagement with PIR.
Isaacs et al. (56)	Australia	Community	Report on the views and experiences of stakeholders on the PIR initiative in Gippsland	Qualitative	Descriptive	*N* = 45	Not reported Not reported	Report on the views and experiences of stakeholders on the PIR initiative, and what differences, if any, it made.	The PIR initiative brought hope to the lives of individuals living with SPMI. Some participants said the emotional support they had received and the awareness they had obtained about the various services available was the “difference between life and death.” They spoke of getting back hope from a position of despair. Engaging with PIR has also fast-tracked the recovery journey of some clients. For those whose recovery journey was expected to take longer, PIR improved their ongoing quality of life.
**Rehabilitation and recovery-focused**									
Heatherington et al. (65)	USA	Farm—residential treatment center	Examine clinical and personal recovery and facilitate program improvement	Quantitative	Uncontrolled pre-post	*N* = 259	29.5 (9.1) 68%	Quality of life (Lehman QLS), psychiatric status (BASIS-24), and functioning (GAF).	Significant improvements on all measures (medium effect sizes), including functioning, symptoms, and relational and independent living skills dimensions of QoL; maintenance of treatment gains at 6 months after discharge (and beyond). Paid work (part- or full-time) was reported by 30–50% across the follow-ups.
Markiewicz et al. (64)	Poland	City daycare center	Use neurofeedback (NF) training as the add-on therapy in patients with schizophrenia to improve their clinical, cognitive, and psychosocial condition within a standard rehabilitation program	Quantitative	RCT	*N* = 44 (intervention = 18, control = 26)	Intervention = 37.2 (6.4), control = 36.4 (8.9) 100%	Symptoms (PANSS), psychosocial (BCIS—insight, AIS—illness acceptance), and self-efficacy (GSES).	Significant changes in both groups (symptoms), and intervention only (insight, illness acceptance, self-efficacy); *post-hoc* analyses showed NF was significantly more effective for self-efficacy.
Nibbio et al. (62)	Italy	Rehabilitation center	Assess feasibility and effectiveness in a real-world care setting, of a practical integrated rehabilitation program (pharmacological treatment, cognitive remediation (CACR), and social skills training) for people with schizophrenia	Quantitative	Pre-post	*N* = 72	39.08 (11.98) 65%	Primary: Real-world functional outcomes (GAF). Secondary: Psychiatric symptoms (PANSS and CGI).	Integrated treatment protocol is feasible and has a positive impact on functional (GAF: large effect size) and clinical outcomes. Low attrition suggests good tolerance and appreciation of treatment.
Parker et al. (60)	Australia	Five Community Care Units (CCUs)	Examine factors predicting improvement in outcomes among CCU consumers	Quantitative	Retrospective cohort	*N* = 501	35.7 70%	Primary: Mental health and social functioning (HoNOS). Secondary: Disability (LSP-16) and accommodation instability.	43.0% showed reliable and clinically significant (RCS) improvement in mental health and social functioning. No changes in disability or accommodation instability. Higher baseline impairment in mental health and social functioning and longer episodes of CCU care increased the likelihood of RCS improvement in mental health and social functioning.
Parker et al. (58)	Australia	Three community Care Units (CCUs)	Explore consumers' experience and understanding of a CCU 12–18 months after service entry	Qualitative	Longitudinal mixed-methods evaluation using semi-structured interviews	*N* = 15	Residents in: clinical model = 29.4 (3.91), integrated model = 33.00 (2.92) Clinical model 80%, integrated model 70%	Consumers' experience and understanding of a CCU after a period of residence.	Seven overarching themes: the first concerned understanding of the CCU as being “about people with mental illness recovering;” other themes included relational [“Staff (can) make a big difference,” “Co-residents (can) provide a good little community,” and “Providing a sense of community inclusion”] and non-relational (“An environment providing opportunities for activity engagement,” “Supportive processes to increase one's independence”) aspects. Experience of care with the integrated staffing model was generally comparable to the traditional clinical staffing model.
Rees et al. (59)	Australia	Three community Care Units (CCUs)	Understand the use of Action Over Inertia (AOI) in CCU settings from the viewpoints of group participants and facilitators	Qualitative	Naturalistic case study informed by an interpretive standpoint	*N* = 10 (AOI group participants = 5; group facilitators = 5)	AOI participants = 42 Not reported	Experiences of AOI, and its impacts on achieving a sense of recovery, from the perspective of adults with SMI. AOI group facilitators' views and experience of facilitating the intervention.	Two overarching themes are “Making Change” and “Facilitating Change.” For AOI group participants, making change involved finding it hard to get themselves going, and recognizing the importance of doing so; AOI group participation enabled recognition of the value of meaningful activities and that doing things brings a sense of hope and recovery.
Sanches et al. (63)	Netherlands	Community	Establish the effectiveness with which the Boston University Approach to Psychiatric Rehabilitation (BPR) improves the level of social participation in people with SMIs in the Netherlands	Quantitative	Multi-center two-parallel-arm RCT	*N* = 188 (intervention = 98, control = 90)	39.9 (11.3) 58%	Primary: Social participation [employment (SFS_OE)], total hours in paid or unpaid employment over 6 months, and Dutch National Societal Participation Ladder). Secondary: QoL (MANSA), personal recovery (RAS), self-efficacy (GSES), psychosocial functioning (SFS), disability (GAF), and symptoms (BPRS).	Social participation improved significantly, but BPR did not improve social participation more effectively than the active control condition. Previous employment and baseline psychiatric symptoms consistently predicted the primary outcome. The rate of improvement did not differ between the conditions for any of the secondary outcome measures.
Thomas et al. (61)	Australia	Community-based sub-acute residential service	Examine changes in step-up and step-down clients' symptoms and functioning after admission to a sub-acute residential recovery-focused program, from the perspective of clients and service providers	Quantitative	Uncontrolled pre-post	*N* = 41	36.5 (11.4) 51%	Symptoms and functioning (BASIS-32, HoNOS), life skills functioning (LSP-16), and quality of life (AQoL-8D).	Improvements between admission and exit in relation to self and others, psychosis, daily living, role functioning, depression, or anxiety symptoms (client measures, small to large effect sizes). Gains in self-care (medium effect size), level of symptoms (step-up—large effect size, step-down—medium effect), and presence of social problems (step-up clients only, large effect; service provider reports). At 3 months after discharge, clients rated no change in symptoms and functioning, i.e., gains were maintained.
**Social and community connection-focused**									
Agrest et al. (67)	Chile	Consumer's home	Evaluate from a user perspective the feasibility, acceptability, and applicability of a community-based psychosocial intervention in urban settings in Latin America	Qualitative	Thematic analysis of semi-structured individual interviews	*N* = 15	40.1 66.6%	Users' perspectives regarding the *in vivo* approach and the strategy of task-shifting community-based mental health support to PSWs and community mental health workers.	*In vivo* services were viewed as a road to recovery; most appreciated the flexibility of being seen at home whereby workers had more understanding of their situation and were better able to provide support. Others are worried about being seen at home and being stigmatized by neighbors regarding their use of mental health services.
Agner et al. (70)	USA	Clubhouse	Provide a culturally responsive perspective on wellness and illustrate the value of Clubhouses as a space for mental health recovery and transformative change	Qualitative	Photovoice/ participatory research	*N* = 43 (clubhouse members = 37 and staff = 6)	Clubhouse members = 52.6, staff = 50.5 Clubhouse members = 68%, staff = 33%	Pilinaha, a Native Hawaiian framework for health.	Themes included “Connection to Place,” “Connection to Community,” “Connection to Better Self,” and “Connection to Past and Future.”
Gumber et al. (72)	USA	Community	Examine the relative contribution of individual member characteristics, community supports, and the clubhouse environment in accounting for variation in members' reports of social integration within the clubhouse and the larger community	Quantitative	Descriptive cross-sectional survey	*N* = 92 (*n* for intervention and control groups not reported)	46.4 (11) 54%	Social integration within clubhouse (CIS), social integration within community (ESIS), perceived familial support (FAPS, ESIS), mental health symptoms (CSI), and self-esteem (RSES).	Adults who spent more time at the clubhouse and viewed the clubhouse as having a more practical orientation reported feeling more integrated into the social aspects of the clubhouse; 42% of the variance in participants' reports of social integration outside the clubhouse with non-consumers was accounted for by participants' reports of self-esteem and perceived family support. Self-esteem accounted for significant variance in perceived social support in the community, but not social integration within the clubhouse. Greater family support was associated with high social integration in and outside the clubhouse.
Jensen et al. (33)	Denmark	Two social psychiatric residences and one community mental health center	Conduct an in-depth investigation of participants' lived experience of personal goal-setting during their participation in an illness management and recovery (IMR) program	Qualitative	Descriptive phenomenological study	*N* = 15 (CMHC = 7 and residential homes = 8)	Range = 30–72 33%	Experience of goal-setting and impact of IMR on the personal recovery process, including what changed regarding the view of self and the view of illness.	Pursuing personal goals broke barriers and participants saw small aspects of their everyday lives change. Although participants learned how to structure the breakdown of personal goals into smaller short-term goals during IMR, they often stopped making progress toward their short-term goals.
Kidd et al. (66)	Canada	Transition from hospital to community	Assess the effectiveness of a brief, transitional, peer support intervention on community functioning for individuals diagnosed with schizophrenia when leaving hospital	Quantitative	RCT	*N* = 110 (intervention = 41, brief version = 23 and control = 46)	34.6 61.7%	Primary: Community functioning (MCAS, cMCAS). Secondary: Symptomatology (BSI), community integration (CIS), personal recovery (PROM), quality of life (SLS), and social support (SSS).	No difference across the three groups in community functioning or any other outcome.
Pernice et al. (71)	USA	Community	Investigate the motivations and reasons of people who seek support from voluntary recovery communities, like clubhouse	Qualitative	Semi-structured interviews, cross-sectional design	*N* = 143	Not reported 54%	The reasons people come to the clubhouse, including what would be different if there was no clubhouse and in what ways the clubhouse assists with recovery.	People accessed the clubhouse for social connection/to reduce isolation; something to do; symptom management; gaining skills; and enjoyable culture. A group of people with SMI seeks out voluntary recovery communities because they see benefits of wellbeing (including but not limited to symptom reduction) and happiness through social systems—social connection, having something to do and look forward to, gaining skills through work-ordered day and structured activities and the enjoyable culture of the centers.
Salzer et al. (69)	USA	Community mental health centers	Examine the effectiveness of peer-delivered core services of Centers for Independent Living (CILs), which include advocacy, information and referral, skills training, and peer support	Mixed methods	RCT	*N* = 99 (intervention = 50 and control = 49)	48.7 (8.8) 53%	Community participation (TUCPM), recovery (RAS), empowerment (ES), and quality of life (QoLI).	No significant differences were found in repeated measures analyses. *Post-hoc* analyses did show some positive results for those in the CIL condition. Specific outcome measures were not revisited in the results.
Varga et al. (68)	Hungary	Community	Examine prospective changes in social cognition and functional outcomes in two groups of schizophrenic patients involved in CM and community-based club (CC) compared to a matched, TAU group of patients	Quantitative	RCT	*N* = 75 (CC = 26, CM = 26, control = 23)	39.6 (8.4) 50%	Psychopathology and psychosocial functioning (PANSS and GAF).	Functional outcomes improved significantly in the CC as well as in the CM groups, in contrast to the TAU group.
**Supported accommodation**									
Bitter et al. (77)	Netherlands	Sheltered and supported housing	Investigate the effectiveness of the comprehensive approach to rehabilitation (CARe) methodology for people with severe mental illness on their quality of life, personal recovery, participation, hope, empowerment, self-efficacy beliefs, and unmet needs	Quantitative	Cluster RCT	*N* = 263 (intervention = 152 and control = 111)	50.8 (14.3) 65%	Primary: Quality of Life (MANSA), social functioning (SFS), personal recovery (Mental Health Recovery Measure, MHRM). Secondary: Empowerment (DES), hope (HI), and self-efficacy (MHCS).	Did not lead to more improvement in clients' quality of life, personal recovery, and social functioning. Clients in both groups improved on quality of life. At T1, a small to medium significantly different change score between the intervention and control group was found on both quality of life in favor of the intervention group. At T2, no differences were found. No improvement in hope, or empowerment. or self-efficacy.
Bochicchio et al. (79)	USA	Supported housing	Explore how people with SMI living in supportive housing perceived receiving support from peer and non-peer providers for their physical health	Qualitative	Interviews, grounded theory	*N* = 28	49.8 (9.3) 50%	Open-ended. questions asked about comfort, relationships with clinicians, and engagement with peer workers.	Participants viewed their relationships with peer and non-peer workers positively but described differences in the approach to practice, power dynamics present, and how they identified with each provider. Peer workers were described in terms of increasing hope and understanding.
Dunt et al. (73)	Australia	Supported housing	Estimate Doorway participants' outcomes for housing, health, and mental health service use for people with SPMIs and precarious housing, referred from the public mental health system	Quantitative	Quasi-experimental study design with a comparison group, adjusted for 10 potential confounders	*N* = 237 (intervention = 157 and control = 80)	Intervention = 34.7 and control = 37.3 Intervention = 57%, control = 69%	Secondary: clinical recovery (HoNOS).	There was a significant, positive Doorway effect on clinical outcomes (positive effects on HoNOS scores—social subscale and overall scale).
Mejia-Lancheros et al. (74)	Canada	Community-based study center	Identify distinct wellbeing trajectory profiles over a 6-year follow-up period among adults experiencing homelessness and mental illness	Quantitative	Pre-post	*N* = 543 (intervention = 292 and control = 251)	40.3 (11.7) 68%	Subjective wellbeing (Lehman's 20-item QOL interview) and community functioning (MCAS).	HF interventions improve the longitudinal wellbeing profiles (subjective wellbeing, community functioning) of homeless people with mental health problems over a 6-year follow-up.
Rhenter et al. (76)	France	Community services	To examine what constitutes recovery from the patient's point of view and what recovery trajectories look like within the French context	Qualitative	Semi-structured interviews	*N* = 36 (intervention = 24 and control = 12)	Not reported	Recovery experiences before and following the move to HF service.	Stable housing is “a refuge” that prompts reflection on the past and hopes for the future. Initial honeymoon period is often followed by difficulties in sustaining positivity. Challenges to the success of HF on client trajectories include finding a balance between protection and risk and interrupting downward spirals. Effects of HF on recovery are considerable, but insufficient to prevent negative trajectory.
Somers et al. (75)	Canada	Scattered, congregate, or other housing	Determine if “congregate” HF (CHF) and Scattered-site HF (SHF) would be associated with a greater percentage of time stably housed as well as superior health and psychosocial outcomes over 24 months compared to TAU	Quantitative	RCT	*N* = 297 (intervention 1 = 107, intervention 2 = 90 and control = 100)	Intervention 1 = 40.0 (11.6), intervention 2 = 39.5 (10.8) and control = 39.5 (11.2) Intervention 1 = 77%, intervention 2 = 74%, control = 71%	Secondary: Severity of disability (MCAS), community integration (CIS), psychiatric symptom severity (CSI), quality of life (QoLI-20), and recovery (RAS-22).	Secondary outcomes favored CHF but not SHF compared to TAU. The mean change in MCAS score (severity of disability) from baseline to 24 months was significantly different between TAU and CHF participants but not between TAU and SHF participants. Mean change from baseline to 24 months did not differ significantly between SHF and TAU for community integration on psychological subscales, and psychiatric symptom severity. Mean change from baseline to 24 months was significantly greater in CHF compared to TAU for psychological, community integration and recovery.
Zarchev et al. (78)	Netherlands	Sheltered housing, independent living, and family-supported living	Identify differences in prevalence and incidence of crime victimization in sheltered housing compared with living alone or with family	Quantitative	Cross-sectional survey embedded in the Victimization in Psychiatric Patients study	*N* = 956 (*n* for intervention and control groups not reported)	44.7 (10.4) 64%	Prevalence of crime victimization and the number of incidents in the past year (crime victimization scale of the DCVS).	Victimization prevalence was highest among residents in sheltered housing (50.8%) compared with persons living alone (43%) or with family (37.8%). Incidence was especially high for men, people with comorbid post-traumatic stress disorder, and those with high levels of education. However, women reported less victimization in sheltered housing than living alone or with family, if they also reported drug or alcohol use.
**Vocational support**									
Christensen et al. (81)	Denmark	Early-intervention teams or community mental health services	Investigate the effects of individual placement support vs. independent placement support with enhancements vs. service as usual on a population of individuals with severe mental illness in Denmark	Quantitative	RCT	*N* = 720 (intervention 1 = 243, intervention 2 = 238 and control = 239)	32.8 (9.9) 62%	Secondary: Clinical recovery (SANS, SAPS, SF-12). Social functioning (Personal and Social Performance Scale), self-esteem (Rosenberg Self-esteem Scale), and self-efficacy (General Self-efficacy Scale).	No difference between the groups on non-vocational (secondary) personal, functional, or clinical recovery outcomes.
Gal et al. (83)	Israel	Vocational settings in the community—employment, sheltered workshops, or vocational support centers	Compare patient-reported outcome measures between consumers and service providers for people in 3 different vocational services—IPS, sheltered workshops, and vocational support centers that focus on skills training and leisure	Quantitative	Cross-sectional descriptive study	*N* = 4,216	46.7 (12.3) 57%	QoL—housing, relationship, social, family relations, leisure activity (MANSA), insight (3/15 items from IMR), and effects of symptoms on daily functioning (SDS-3).	92% of participants perceived themselves as a worker regardless of service type. According to providers, IPS is associated with better functioning and illness management but not QoL. For consumers, IPS was associated with better functioning only. Percentage of disability is lower for people receiving IPS (52.5+-15.9), compared to vocational support (55.4+-15.9) or sheltered workshops (56.2+-18.5). QoL rated by consumers found no difference between services. Overall functioning scale indicated significant differences for IPS compared to other services for both consumers and staff. IMR had significant positive differences for IPS as rated by staff but no significant differences rated by consumers.
Gammelgaard et al. (82)	Denmark	Community MHS	Investigate how IPS and employment influence recovery in persons with severe mental illness	Qualitative	Phenomenological hermeneutic study	*N* = 12	Range 28–59 75%	The aim of the study was to describe how IPS and employment may influence recovery as experienced by persons with SMI.	IPS and competitive work have an impact on personal recovery. Some participants considered increased self-esteem and skills to change life patterns as components involved in recovery. Participants spoke of being part of society and having supportive, collaborative relationships with professionals as important to recovery. IPS and employment contain elements that can be identified by the five personal recovery processes described by CHIME. May decrease depressive symptoms, with no impact on psychotic symptoms.
McGurk et al. (86)	USA	Prevocational community psychiatric service	Evaluate the feasibility of implementing an empirically supported cognitive remediation program in routine rehabilitation pre-vocational services at two sites	Quantitative	Pre-post feasibility trial	*N* = 83	Intervention = 39.0 (8.6) and control = 36.4 (10.0) 65%	Clinical recovery (PANSS).	Participants improved marginally in clinical recovery. Thinking Skills Work participants improved marginally significantly more in overall symptom severity and the activation subscale of the PANSS compared to participants in the Enhanced Vocational Rehabilitation program. There were no differences between the groups on the other PANSS subscales.
Miles et al. (80)	Australia	Not-for-profit employment service	Evaluate the effectiveness of the Employ Your Mind program in improving cognitive skills and psychosocial outcomes relevant to employment and community engagement in individuals with SMIs	Quantitative	Pre-post	*N* = 32	40.0 (12.1) 56%	Wellbeing (WEMWBS), recovery [The Questionnaire about the Process of Recovery (QPR)], and quality of life (AQoL-6D).	There were trends toward improvement across all psychosocial measures. Participants reported significant increases in positive mental wellbeing, mental health QoL, and overall QoL. Improvements were also seen in other AQoL subscales (independent living, relationships, and coping). Increased social adjustment and self-reported recovery, but these did not remain significant after correction for multiple comparisons.
Rodríguez-Pulido et al. (84)	Spain	Community MHS	Investigate the effects of cognitive remediation (CR) training with IPS in people suffering from SMI in the European population	Quantitative	RCT	*N* = 47 (intervention = 23 and control = 24)	Not reported 68%	Symptoms (PANSS). For people with schizophrenia, PANSS was administered in all its subscales, while for the rest of the sample, the general psychopathology scale was administered.	Symptomatology did not significantly change in any of the three measured components—positive scale, negative scale, and psychopathology.
Schneider et al. (85)	UK	Community MHS	Explore whether IPS outcomes could be enhanced with work-focused counseling	Quantitative	Pragmatic RCT pilot	*N* = 74 (intervention = 37 and control = 37)	Intervention = 30.5 and control = 29.5 70%	Secondary: Self-esteem (RSES), Quality of Life (EQ-5D); health and wellbeing (SF-12).	No difference was found at an individual level for most of the secondary outcomes between baseline and 6 months and baseline and 12 months. Mean scores for self-esteem were not significant. Individuals perceived their health state score on the EQ-5D to worsen over time. Any additional benefit of counseling over IPS alone could not be ascertained, due mainly to the high drop-out rate.
Twamley et al. (87)	USA	Community	Test a 12-week, manualized, Compensatory Cognitive Training (CCT) intervention targeting prospective memory, attention, learning/memory, and executive functioning in the context of supported employment for people with SMI who were seeking work	Quantitative	RCT	*N* = 153 (intervention = 77 and control = 76)	43.7 (11.7) 57%	Secondary: Social Skills Performance Assessment (SSPA). Symptom severity measures (Hamilton Depression Rating Scale [HAM-D], Positive and Negative Syndrome Scale (PANSS), the Independent Living Skills Survey (ILSS), and the Quality of Life Interview (QOLI).	CCT in the context of a supported employment program for people with SMI confers an initial benefit on depressive symptoms and subjective quality of life. Across intervention groups, participants with a diagnosis of a mood disorder improved more on social skills (SSPA) and symptoms of psychosis (PANSS positive and negative).

ACT, Assertive community treatment; CBT, Cognitive behavioral therapy; CHF, Congregate Housing First; CM, Case management; CMHC, Community mental health Center; CCU, Community care unit; FACT, Flexible assertive community treatment; HF, Housing First; ICM, Intensive case management; IMR, Illness management and recovery; IPS, Individual placement and support; MHS, Mental health service; PSW, Peer support worker; SMI, Severe mental illness; SPMI, Severe and persistent mental illness; TAU, Treatment as usual.

**Table 4:** Content and delivery of the models of care.

**References**	**Delivery component (delivery personnel, delivery mode, time/session numbers, and group/individual/virtual)**	**Content component (content overview)**
**Goal-focused**		
Cook et al. (32)	A managed behavioral health “carve-out” that offered an integrated, single system of care overseen by the managed care company value options.	Participants developed person-centered plans for recovery as mandated by the federal Centers for Medicare and Medicaid Services. They created individual budgets with line items for the purchase of services and goods corresponding to plan goals, which were reviewed and approved by program management.
Enrique et al. (31)	Internet-delivered, self-management intervention for bipolar disorder offered along with TAU at two secondary-care services in Ireland. Supported by on-site clinicians who were to provide 6 reviews to their clients over 10 weeks.	Bipolar toolkit includes 4 core modules, aiming to promote personal recovery and quality of life. The modules are (1) the facts about Bipolar; (2) Bipolar and Me; (3) Relationships; and (4) Sleep. Clinicians were trained during a 3-h workshop.
Jensen et al. (33)	One hour weekly/bi-weekly session provided by one therapist trained in CBT and one mental health experienced co-therapist (not a therapist), mostly in people's homes. Both were trained in manual, family work, and cultural awareness/cross-cultural working.	Based on Barrowclough and Tarrier's CBT model of family intervention and adapted for Afro-Caribbean backgrounds. Participation included 10 sessions focused on engagement, shared learning, communication, problem-solving and stress management, and staying well.
Vizzotto et al. (29)	30 × 90-min sessions over 15 weeks, plus activity-based homework assignments practice in a real-world context. Focus on 3 groups of functional activities—food preparation, money management, and reading/writing/information seeking/computer literacy.	Occupational goal intervention involves a structured cognitive strategy-learning intervention (raises awareness of, and targets, executive functioning deficits) to support the performance of complex everyday activities. Focus on choosing meaningful activities and debriefing on activity performance.
**Integrated community treatment (including case management)**		
Corrigan et al. (39)	Peer Navigators provided support to participants at least once weekly. Meetings occurred as often as five times a week.	Peer Navigator Program developed for African Americans was redeveloped by community-based participatory research (with Latinos) for their population. Designed to assist with meeting individual needs while traversing a complex health system.
Errichetti et al. (41)	At least two visits with a primary care provider and at least one visit with a chronic care nurse or dietician.	Integrated care model based on the Wagner model for effective chronic illness care featuring a delivery system linked with complementary treatment and services, sustained by productive and synergistic interactions between multidisciplinary care teams and patients.
Gelkopf et al. (36)	Intervention was the newly established strength-based case management service (SBCM) in addition to regular psychiatric rehabilitation services (PRS). Included family focus. Caseload maximum of 32:1. Case managers engage in regular SBCM training and supervision. Case manager availability and intensity and other practical details of TAU-PRS were lacking.	SBCM promotes active engagement of clients in defining and attaining personally meaningful goals. Includes assistance in selecting and utilizing services and natural community resources that are most likely to help SBCMs' role: work from consumers' own goals, liaise with other services, e.g., integrated employment, and other rehabilitation services. TAU-PRS includes education, employment, housing, and family support.
Li et al. (38)	Face-to-face via home visits.	Home nurses have several tasks and goals, including (1) establishing an alliance with their patients; (2) assessing patient-care needs; (3) considering both medical and social-care practices; (4) addressing patients' self-management of medication and their daily tasks; (5) providing crisis intervention; and (6) coordinate resources.
Mahlke et al. (35)	One-hour, one-to-one peer support sessions delivered in inpatient and community settings (mean 12.2 (SD 9.6) sessions). Participants and peer supporters met 4–26 times over 6 months.	Peer support model included practical support with everyday life, helping to endure and understand crises, sharing ideas about planning and recovery, providing information, and mediating conflicts with clinicians or family.
O'Connell et al. (42)	Recovery mentors (RMs) provided one-to-one sessions independent of community mental health service. Mean number of sessions was 13 (SD 11.3); mean hours was 24.9 (SD 18.8), over 9 months.	Team of 8 RMs trained to provide peer support independent of community mental health service. Used team-based practice and recovery principles. Focus on recovery promotion, identifying assets, strengths, and goals of the mentees, and links with local resources. RM training included professional and personal boundaries safety, cultural competence, and gender- and trauma-informed care. Weekly supervision.
Palmer et al. (34)	Co-design intervention included stages of information gathering, training and 5 design/collaborative meetings, and an implementation phase.	An adapted/truncated version of experience-based co-design was used with consumers and carers to design community support practices. Included the development of locally tailored action and implementation plans for service improvement, including communication and information flow, user involvement in service design, and improved spaces.
Sahin et al. (43)	Community mental health centers with the aim of keeping patients in active treatment as an outpatient.	Psychoeducation, collective social activities, daily skill therapies, group therapies, and phone call reminders.
Soberay et al. (40)	The integrated health home, the Hope Health and Wellness (HHW) Clinic, provides comprehensive primary and behavioral health services. The HHW clinic was co-located with a fitness center, available at no cost to clients. Coordination with a personal trainer in the fitness center occurs weekly or as needed with case conferences to support clients' health and fitness goals. Data analysis was applied to consumers who consented and were enrolled in the service for 18 months or more. No other reports of actual service use for participants are included.	The team included: behavioral health provider/licensed psychologist (supports individualized health and wellness plans: health goals and leads wellness groups), care coordinator (support with transportation and other psychosocial needs, and navigating specialty care referrals), referral coordinator (integrates records between systems, supports patients in getting connected to the clinic), peer specialists (provide wellness groups or peer support during and outside of clinic), a primary care provider, and a medical assistant.
Valls et al. (37)	12 weekly group sessions, 90 min each, implemented in a closed-group setting, over 3 months, via an outpatient clinic. Groups had 10 to 14 participants each.	Psychoeducation for consumers combined with a session for family members only. Content also related to healthy lifestyle, mindfulness training, and strategies for cognitive and functional enhancement.
**Intensive case management**		
Albers et al. (48)	Treatment provided by a multidisciplinary FACT team with a shared caseload. Intervention participants also received the Victoria intervention through a) initial face-to-face individual session, and b) face-to-face sessions with worker and family/significant others. The number of sessions varied, they lasted 15–60 min.	Intervention: FACT team provided individual case management and assertive community treatment (FACT and the Boston University Approach to Psychiatric Rehabilitation, BPR). Plus, the Victoria (victimization-informed intervention for professionals) intervention: (1) explore the victimization experience and its impact on life domains with the client, (2) identify the most negative experiences related to social participation, (3) identify context—why the person engaged in the situation leading to victimization and shift from negative to positive stance, and (4) future plans. TAU: FACT and BPR.
Barakat et al. (46)	Intensive Home Treatment (IHT) teams are multidisciplinary and provide intensive care at least twice a week and continue until the crisis is resolved, for an average duration of 6 weeks. TAU comprised either specialized mental health hospital care or other less intensive outpatient care (i.e., two times a week or less).	IHT teams offer psychiatric treatment, emotional and practical support, and psychoeducation for the patient and their relatives and focus on improving problem-solving and everyday skills. TAU treatment depended on the severity of symptoms, presence of danger, housing, and availability of a support system.
Ben-Zeev et al. (47)	ACT: delivery details not described, other than as an intensive team-based treatment model. Intervention group received ACT and daily recovery-oriented texting exchanges with a trained community-based mental health worker over 12 weeks. Texting is available Mon–Fri 9–5 only, but ACT is available for extended hours.	ACT: provides comprehensive psychiatric, rehabilitation, and support services in the community. Texting was used to augment ACT in the intervention group. Recovery-oriented texts included appointment and prescription reminders, information (e.g., psychoeducation, links to resources), cognitive techniques (e.g., restructuring dysfunctional beliefs about voices), self-monitoring of symptoms, relaxation techniques, social skills training, supportive messages, and *in vivo* support.
Blankers et al. (49)	TAU was FACT (low intensity, flexible adaptation of ACT; multidisciplinary team uses a flexible switching system to provide intensive care via a shared caseload approach at any time or day). Intervention participants also had a computer, Internet, and webcam installed at home and Skype to talk to staff, on average 2–3 times a week and as needed during office hours.	Access online to psychoeducative videos, a leisure activities bulletin board, an agenda for scheduling appointments with the psychiatric nurse, and a web forum to establish contact with other patients. Participants also had access to Skype to talk to clinicians.
Clausen et al. (51)	The assertive community treatment (ACT) model is a multidisciplinary, team-based, and intensive, service delivery program.	Psychosocial and outreach services with a strong focus on improving their patients' abilities to achieve and sustain an independent life in the community. No further details were given, but the intervention appears to be standard ACT.
Incedere et al. (53)	'Hybrid' case management delivered by the same case manager. Support was offered 9–6 pm weekdays and by telephone 24 h a day, 7 days a week. Individual and group sessions, depending on focus.	Access to Community Mental Health Center, home visiting, individual counseling, psychoeducation, psychosocial skills training group, family psychoeducation, and supported employment.
Iyer et al. (45)	Regular care comprised primary care (family physician and/or community health and social service centers) or secondary care (hospital-based outpatient appointments with psychiatrists and allied staff). Few details are given about the delivery of Early Intervention (EI). Described as case management.	Regular EI (first 2 years) offered CM, medication, and psychosocial interventions. Extended EI focused on relapse prevention, treatment adherence, crisis and substance abuse management, and functional recovery. Psychoeducation, multiple family group therapy, peer support, CBT, and IPS also available. Content of regular care not described.
Klodnick et al. (54)	Multidisciplinary team approach blending ACT and the Transition to Independence Process (TIP) models. Intensive outreach and support: participants are seen multiple times per week, with staff-to-participant ratios ~1:10. Text reminders, face-to-face contacts, and weekly activity groups are offered. Services are available 24/7.	Goal-orientated, collaborative, recovery-based approach. Art therapy, movement, and mind-body-based practices, IPS, supported education, Dialectical Behavior Therapy, and CBT were provided.
Nugter et al. (50)	FACT multidisciplinary teams offered two levels of care: individual case management for most patients, and full ACT with shared caseload and assertive outreach when needed. FACTs: moderate (1 team) to high (2 teams) fidelity.	No description of the content of care was given, but the model was assumed to be standard FACT.
Siskind et al. (44)	Time-limited intensive case management provided by a multidisciplinary team, including a maximum staff-to-consumer ratio of 1:12. Minimum weekly contact and frequent psychiatric medical review.	Medical review, illness self-management, and group and individual sessions including cognitive remediation, social cognition, CBT for psychosis, sensory modulation, yoga, swimming, walking, and cooking skills.
Vidal et al. (52)	“Intensive” care in the community is provided during office hours. Time-unlimited. Staffing is not described except that “no substance misuse worker was engaged.”	Content not described other than the aims of the team were to engage patients regarded as refractory to usual care and work toward outpatient step-down.
**Partners in recovery care coordination**		
Banfield et al. (57)	Support worker and service linkage.	Tailored, wrap-around care to people with SPMI and complex care needs that had not been adequately addressed. Designed to integrate community health and human services. Comprised a consortium of local organizations and service providers.
Hancock et al. (55)	Support worker and service linkage.	Partners in Recovery services were established to support individuals with SPMI by creating service linkages to address unmet needs to facilitate recovery. Services were delivered through the new role of “support facilitator.”
Isaacs et al. (56)	Care coordination via in-person support facilitators (SFs). Facilitated by the SF, care teams met regularly with the client to monitor progress.	Giving the client the first voice, the care team developed a care plan for the client. After approval of the plan by the client, each member of the care team delivered their component of the plan in collaboration with other care team members.
**Rehabilitation and recovery-focused**		
Heatherington et al. (65)	Non-profit residential treatment center on a 700-acre working farm with team-based work programs. Guests work for 30 h/week, and regular meetings are held with mental health counselors, support groups, and a transition counselor. The median stay was 10 months.	Milieu treatment is designed to create a recovery-oriented environment in which individuals can discover their strengths and interests while clinical needs are addressed. Interventions include counseling, medication, recreation, community involvement, and a work program to develop skills needed for psychosocial rehabilitation.
Markiewicz et al. (64)	Neurofeedback (NF) training 2x/week for 3 months; at least one teamwork rehabilitation session is offered daily.	NF uses the galvanic skin response method—as an add-on therapy to regular clinical management, psychopharmacotherapy, and standard rehabilitation (social activities to build social competence, personal acceptance, and independence).
Nibbio et al. (62)	Computer-assisted cognitive remediation (CACR) individually 45 min × three times per week for 6 weeks, manualized group Social Skills Training (SST) 45 min × twice weekly for 8 weeks with daily feedback sessions.	In addition to standard care (case management, social groups, and leisure activities), the program included CACR (using Cogpak) followed by SST focused on communication, conversation, assertiveness, and friendship skills.
Parker et al. (60)	Clustered, independent living units providing time-limited, 24-h, rehabilitation support.	Residential clinically oriented rehabilitation support focused on improving multiple aspects of personal functioning—primarily living skills development and community integration—in the context of overall mental health.
Parker et al. (58)	Three CCUs provide 24-h, time-limited (6–24 months) rehabilitation support: 2 with an integrated (predominantly peer workers) and 1 with a clinical (predominantly nursing) staffing model.	Intensive recovery-oriented rehabilitation program focused on living skills development and community integration. Available therapeutic interventions include cognitive behavior therapy, cognitive remediation, and social cognitive interventions.
Rees et al. (59)	Group-based manualized Action over Inertia (AOI) delivered by two facilitators, over 5–8 sessions. CCU care was delivered by a multidisciplinary team over 24 h, 7 days per week, in clustered 2–3 bed self-contained units.	AOI is a time-use intervention to build activity patterns that enable fulfilling lives irrespective of the presence of ongoing mental ill-health. Provided as an enhancement to CCU clinical care and rehabilitation support.
Sanches et al. (63)	Delivered by 28 Boston University Approach to Psychiatric Rehabilitation (BPR) trained social workers, nurses, or employment specialists. Minimum one session fortnightly. Fidelity to BPR was assessed for two-thirds of providers; 55 practitioners of similar backgrounds, but not BPR trained, provided active control condition (ACC). Minimum one session fortnightly.	BPR was designed to address housing, education, work, and social contact goals. BPR is a 4-phase intervention—exploring goals, choosing goals, setting goals, and keeping goals. Goal and pace were self-directed. ACC participants were proactively offered support with rehabilitation goals.
Thomas et al. (61)	Five-bed facility with 24-h staffing, offering individual support for up to 3 months. Service is operated in a partnership between a non-government mental health advocacy organization and the health department. Step-up clients enter from the community and step-down clients transition from the inpatient unit.	Provides accommodation, psychosocial educational groups, and other activities designed to support recovery. Support ranges from illness management and relapse prevention strategies to the teaching of life skills.
**Social and community connection-focused**		
Agrest et al. (67)	Three phases were delivered by a community mental health worker and PSW pair over about 9 months. Delivered in people's homes.	Phases: 1. initiation (1–3 months), support provided to consumers to enable connection to people and community agencies that will provide primary support. 2. Try-out (4–6 months), monitoring of the consumer to ensure the strengthening of social support networks. 3. Transfer of care (7–9 months) gradual termination of Critical Time Intervention-Task Shifting services.
Agner et al. (70)	Face-to-face meetings at each Clubhouse.	Sessions involved sharing photographs from each question and analyzing each photograph as a group.
Gumber et al. (72)	Psychological rehabilitation clubhouses.	Clubhouse work-ordered day.
Jensen et al. (33)	Participants attended the 9-month course, a minimum of 20 sessions, facilitated by psychologists or experienced mental health professionals with a minimum 3-day training in IMR. Delivered in 2–3 weekly groups in community residential homes for those living there, or at CMHC. Weekly individual follow-up in between for consultation, advice, and support. Use of personal IMR tracking sheet updated jointly every week.	IMR, which included a curriculum-based rehabilitation program developed by Mueser et al. (93). Intervention included a goal-oriented illness management program (addressing biological factors, building social support, and enhancing coping) for people with SMI to achieve clinical and personal recovery. Implemented according to IMR fidelity scale.
Kidd et al. (66)	Intervention (WB full) was 1–2 inpatient peer support contacts of < 1 h in the 1–2-week period before discharge. A “welcome basket” (budget $40) was supplied to home after discharge, along with weekly PSW contacts of 1–2 h, for 1 month post-discharge. Brief intervention (WBbr) included 1–2 inpatient peer support contacts while inpatient, and one visit at home.	A “welcome basket” of consumables supplied to home after discharge containing: staple supplies, plants, coupons for nearby stores, and comfort items. The PSW and client plan tours of the neighborhood to familiarize with local resources (e.g., libraries; parks; and inclusive spaces) and support the client in building confidence in accessing their local communities. Core Cognitive Adaptation Training compensatory interventions (94) also provided, including setting up a calendar, lists of daily activities, signs that prompt recall of tasks, basic organization of living space, and the use of alarms and reminders.
Pernice et al. (71)	Voluntary, member-directed, and staff-supported community for people with SMI. Staffed by social practitioners. Based on a work-ordered day, weekdays 9–5, with recreational activities outside these hours.	Staff create opportunities for meaningful engagement. Programming includes transitional employment, administration, health and wellness programs, culinary food service, and housing assistance.
Salzer et al. (69)	Peer specialists provided one-to-one peer support sessions by phone or in-person. Mean hours of peer support 6.4 (SD 4.7) over 6 months. Peer support specialists had 75 h of training through a registered center and ongoing supervision.	Manualized peer support addressed emotional wellness, physical health, education, employment, leisure activities, housing, meaningful occupation, transportation, drug and alcohol issues, financial needs, civic engagement, and family relationships.
Varga et al. (68)	Case management involved home-based weekly support and an individualized relationship with the case manager. Community club involved continuously available daycare and was supported by staff.	Case management involved individualized care plans, practical support, training, and capacity building, with family included. Community clubs involved social and occupational engagement groups (psychoeducation, social skills, stress management, lifestyle, and music) and connection to the community.
**Supported accommodation**		
Bitter et al. (77)	Intervention delivered via treating team in sheltered housing.	CARe was based on recovery and strength-based approaches to improve social participation. Seven × one or half-day team training sessions plus 4–6 weekly team coaching sessions. Treatment as usual included no specific training in rehabilitation or recovery-based practice.
Bochicchio et al. (79)	The 22-session manualized year-long program, adapted for delivery by peer specialists. Weekly sessions for the first 3 months (core), followed by bi-weekly sessions (transition) for 3 months, and monthly sessions (maintenance) for the remaining 6 months. Face-to-face and telephone support. Also included in-between session check-ins, flexible session formats (group or individual), and make-up sessions.	The Peer-Led Group Lifestyle Balance Program (PGLB) is an adaptation of the Group Lifestyle Balance Program (GLB) derived from the Diabetes Prevention Program. The GLB is a group-based intervention that seeks to reduce the risk of cardiovascular disease and diabetes by improving participants' diet and physical activity.
Dunt et al. (73)	Doorway participants source and choose properties through the open rental market, and subsidies, assistance, advocacy, and brokerage support through their Housing and Recovery Worker.	Doorway extends the original HF model in providing housing support to people with precarious housing. Rental subsidies are provided for people at risk of homelessness who live with SMIs and receive care within Victoria's public mental health system.
Mejia-Lancheros et al. (74)	HF support or ACT plus rent supplement for adults with SMI and a history of chronic homelessness followed for a 2-year period.	HF support, ICM, or ACT plus rent supplement.
Rhenter et al. (76)	ACT included internationally recognized model of intensive multidisciplinary community mental health care (*in vivo* contacts, out-of-hours availability, shared team caseload, etc.). At least weekly visits by ACT team member/s. ACT staff caseloads are limited to 10 per full-time worker.	HF was understood as permanent rented housing in scattered-site tenancies, with subsidized rent. ACT delivers interventions including life skills training, counseling, crisis intervention, and medication supervision. TAU was standard community mental healthcare and housing support.
Somers et al. (75)	Scattered-site HF participants received support in their homes from an ACT team—intensive multidisciplinary community mental health care (small caseloads, *in vivo* contacts, out-of-hours availability, shared team caseload, etc.). “Congregate” HF was mounted in a single vacant building able to house at least 100 occupants in independent suites but without full kitchens; on-site 24x7 supports comparable to ACT were provided.	HF provides support to clients in market housing (i.e., scattered among existing rental accommodations) with a strong emphasis on the promotion of client choice, including sobriety and engagement with treatment. ACT delivers interventions including life skills training, counseling, crisis intervention, and medication supervision. TAU was standard community mental healthcare and housing support.
Zarchev et al. (78)	ACT or ICM support services and rent supplements.	Sheltered housing. No additional data were available on these specifics for those who indicated living in sheltered housing.
**Vocational support**		
Christensen et al. (81)	1:1 meetings with job providers, computer programs, and vocational rehabilitation.	IPS: vocational support per the principles of the IPS model. IPSE: IPS plus cognitive computer training using Computerized Interactive Remediation of Cognition—a Training for Schizophrenia (CIRCUITS), Danish version and training in cognitive coping and compensatory strategies using an adapted version of the Thinking Skills for Work manual. TAU: best available vocational rehabilitation provided by the national job centers.
Gal et al. (83)	Vocational centers offered variable attendance; personnel were not described. Sheltered workshops were available 4–7 h per day on a flexible basis and included rehabilitation directors with mental health training. Supported employment included access to a vocational consultant.	Vocational support centers involve participating in a productive occupation in flexible, non-competitive environments adjusted to a person's abilities—including skills training for employment. Sheltered workshops provided an adjusted working environment, usually a manufacture-like environment, without employer–employee relationships, where consumers are given the opportunity to develop working habits and enhance vocational skills.
Gammelgaard et al. (82)	IPS not otherwise specified.	Evidence-based, recovery-oriented IPS intervention helps persons with SMI achieve competitive employment.
McGurk et al. (86)	Thinking Skills Work (TSW) is tailored at each site and delivered as part of the regular group program. Training of MA-level rehabilitation staff members in TSW (8 h + manual) and supervision.	Vocational services are enhanced by training vocational specialists in recognizing cognitive difficulties and providing job-relevant cognitive coping strategies. TAU: Enhanced Vocational Rehabilitation.
Miles et al. (80)	One-to-two sessions per week, combining computer-based Cognitive Remediation (CR) exercises, individual project work, group reflection/discussion, bridging exercises, and a work experience placement.	Use of qualified CR therapists for cognitive exercises aimed at improving cognitive functioning; procedures to develop problem-solving strategies, and procedures to facilitate transfer to real-world functioning.
Rodríguez-Pulido et al. (84)	Thirty-two sessions weekly for 4 months with neuropsychologists, and 2 rehabilitation sessions per week until work is obtained. Adapted to individual progress after 6 weeks. Feedback to reinforce improvement.	The intervention was embedded in mental health teams with ACT and a Cogpack program for treatment groups, which was individualized according to baseline scores.
Schneider et al. (85)	Work-focused counseling delivered by psychologist at the person's home.	Intervention group received IPS + work-focused counseling. The intervention also included goal-based motivational procedures and CBT designed to address common employment obstacles through developed manuals and self-help materials.
Twamley et al. (87)	Treatment: 12 × 1 h manualized sessions (fidelity assessed and rated at 90% against manual) of a compensatory strategy-based intervention. Delivered individually by masters-level employment specialists (ES) over the first 12 weeks of IPS. Control: IPS + extra sessions with ES.	Four compensatory-based cognitive training modules teaching skills and strategies, addressing prospective memory, conversational and task vigilance, learning and memory, and cognitive flexibility and problem-solving (executive functioning).

ACT, Assertive community treatment; CBT, Cognitive behavioral therapy; CHF, Congregate Housing First; CM, Case management; CMHC, Community mental health center; CCU, Community care unit; FACT, Flexible assertive community treatment; HF, Housing First; ICM, Intensive case management; IMR, Illness management and recovery; IPS, Individual placement and support; MHS, Mental health service; PSW, Peer support worker; SMI, Severe mental illness; SPMI, Severe and persistent mental illness; TAU, Treatment as usual.

## Narrative synthesis

### Overall pattern of findings

This systematic review identified a diverse array of MoCs that seek to improve clinical, functional, and personal recovery outcomes in people who experience SMI. Almost half of the included studies reflected MoCs with a well-established evidence base such as *intensive case management, supported accommodation*, and *vocational support* (10, 95–97). We also identified novel or less established MoCs, which either updated community treatment models, including case management and care coordination (e.g., *integrated community treatment*), or more explicitly addressed participants' personal (and functional) recovery goals [e.g., (32, 67, 68)].

Few of the studies reported on our outcomes of interest (i.e., clinical, functional, or personal recovery) as primary outcomes. Personal recovery was reported as a primary outcome in only four studies (31, 32, 34, 77). The *rehabilitation and recovery-focused* MoC and the *intensive case management* MoC both had the strongest emphasis on clinical outcomes. This may be because they are older MoCs (98, 99). Personal recovery was reported more frequently in newer MoCs (e.g., *goal-focused* and *social and community connection-focused*) and in nearly all models involving peer workers. The findings suggest participants' clinical and functional improvement can be supported by some *intensive case management* and *rehabilitation and recovery-focused* models, adding to the existing evidence of their association with reduced service use (18, 95). We also identified potential for personal recovery to be supported by both these types of MoC, as shown by qualitative studies concerning the *rehabilitation and recovery-focused* MoC, for example (58, 59).

This review identified less *intensive case management* models, often with novel enhancements or better integration of care (see Table 1 for details). These articles reported promising evidence for all three of this review's outcomes of interest (i.e., clinical, functional, and personal recovery) [e.g., (36, 37, 39)]. Our previous systematic review of social interventions for people with SMI (10) suggested that the *supported accommodation* MoC can support consumers' social inclusion. This review also provided some evidence that it could improve personal recovery outcomes such as hope and subjective wellbeing (74, 79). The studies in the *goal-focused* and the *social and community connection-focused* MoCs reported encouraging improvement in some functional and personal recovery outcomes, but we found only limited evidence for the *vocational support* MoC in improving clinical, personal, or functional recovery (80, 83, 86, 87).

Overall, 10 studies reported incorporating peer work. Four belonged to the *integrated community treatment* MoC (35, 39, 40, 42), three to the *social and community connection*-*focused* MoC (66, 67, 69), and one each to *rehabilitation and recovery-focused* (58), *intensive case management* (45), and *supported accommodation* (79) MoCs.

### Factors influencing the findings

The findings of this review should be interpreted with consideration of the potential impact of clinician and researcher bias on the formulation of the research problem, the chosen measurement tools, the interpretation of the results, and the impact of study quality [see Tables 2A, B (Kmet scores)]. Some studies were constructed in a way that lacked a depth of understanding of the complexities of recovery and the needs of participants. Studies often did not report a deeper contextual understanding of the participants' lives, and, particularly in uncontrolled studies, the impact of key mediators, moderators, or confounders such as family support, loneliness, or the impact of stigma.

Overall, the studies typically included clinical, functional, and personal recovery outcomes as secondary outcomes after primary outcomes such as service use. We used a broad interpretation of personal recovery and thus included wellbeing and quality of life outcomes, resulting in all the included MoCs having at least one quantitative or qualitative study that assessed personal recovery outcomes. However, we found that the use of measures specifically focused on personal recovery outcomes was limited, in contrast to other areas of recovery. A small number of measures featured the domains that have emerged from the recovery movement, such as hope and empowerment. These were the *Recovery Assessment Scale* and associated modified versions (RAS) (100), the *Personal Recovery Outcome Measure* (PROM) (101), *Canadian Institutes of Health Information Measuring Patient Experiences in Primary Health Care Survey* (CIHI) (102), the *Mental Health Recovery Measure* (MHRM) (103), *Questionnaire about the Process of Recovery* (QPR) (104), and the *Bipolar Recovery Questionnaire* (BRQ) (105). Only the RAS and modified versions (RAS-R, RAS-D, RAS-DS, and RAS-22) were used in more than one study (32, 34, 39, 47, 52, 55, 63, 69, 75). Thus, when compared to commonly used clinical or functional recovery outcome measures such as the PANSS or GAF, routine focus on personal recovery using established, standardized tools is apparently still in its infancy.

#### Context

The involvement of Lived Experience in the design of the MoCs or the research efforts was not always clear. Few publications reported consumer co-design (34, 70) or consumer involvement in the development or conduct of the study (36, 58, 59, 63), and none were consumer-led. Only Kidd et al. (66) had consumer co-authors, of which there were three. A few studies reported consumer input into the research interview questions (59), the analysis coding framework (58), and consultation to inform the study methodology (63). Peer support was reported in some models across multiple study designs showing beneficial results (35, 79). The clubhouse model was particularly successful in enabling a recovery-oriented environment (70–72).

In addition, few articles took account of relational and family factors, despite these having been shown to be important in the outcome of psychosocial interventions (106). Some of the evidence suggested families and carers were better equipped to provide care when supported. One small uncontrolled study showed significantly reduced “carer burden” (which we took to mean pressure on carers), within an *intensive case management* model incorporating family psychoeducation and supported employment (53). Positive or rewarding impacts of caring were not assessed by any studies, despite evidence that relationships within families may be mutually supportive (107) and culturally significant (70). Furthermore, caring relationships can benefit consumers. An MoC incorporating family involvement (33) showed both personal and interpersonal benefits for consumers. Family support was also associated with better social integration within a Clubhouse (72), echoing the findings of Fossey and Harvey (108).

#### Problems with terminology

Variations in terminology presented problems in understanding and defining the population, the MoC, and the outcomes. Furthermore, to limit the heterogeneity of the target population, we only included consumers experiencing SMI, which posed several challenges, due to the lack of a standardized definition for SMI. Previous conceptions have most often defined SMI in terms of (i) diagnosis; (ii) intensity and chronicity of symptoms; and (iii) complexity of service use needs. Our review defined SMI based on diagnosis, with consideration of the complexity of service needs. Therefore, in articles where our population was comprised of numerous diagnoses, we required that over 50% of the study population experienced schizophrenia, schizoaffective disorder, bipolar disorder, or other severe and enduring psychotic disorders. Articles with participants with unclear diagnoses were excluded. Some articles did not define individuals based on diagnosis, making it challenging to understand who received the intervention [e.g., (72)]. While we understood the potential value of being cautious in not describing people using diagnostic labels, this contributed to challenges regarding who was receiving the intervention and how generalizable the findings may be.

#### Delivery and content of the interventions

We attempted to define MoCs in terms of delivery and content components for this systematic review (23), but the identified studies were still challenging to assess. As an illustration, 32 articles were excluded at the full-text review stage because we could not determine that an MoC was described. The delivery of care in terms of its intensity and availability throughout the week differed between and within the MoC groups, despite our efforts to create meaningful groupings (Table 4). Delivery was typically face-to-face, team-based, and sometimes embedded in existing services [e.g., (48, 53, 66)]. This latter was particularly the case for peer-led or self-directed interventions. There was a typically higher intensity of contact in the *intensive case management MoC* compared with the *goal-focused MoC*. Within the *social and community connection-focused MoC*, the frequency of delivery varied from a mean of 1 h per month (69) to opportunities to participate in a Clubhouse every weekday (71). These variations might help explain differences in outcomes between models, as well as negative outcomes.

The content of interventions within each MoC grouping was also diverse, although generally well-described. Poor descriptions of the content of interventions in some publications may have led to them being excluded from our final sample. Control or comparison groups were also poorly described at times [e.g., (36, 68)]. This was particularly problematic in studies where the intervention was offered as an enhancement to usual care (59, 81, 86).

#### Generalizability

Whether findings are generalizable requires consideration of the study environments, interventions, and participants. All the studies were conducted in high-income countries, and findings may not be generalizable to low- and middle-income countries. Most studies had more male than female participants (see Tables 2A, B). Other factors that could impact generalizability, such as language spoken at home, other service use, and family support, were rarely reported.

## Strength of the evidence

Only two of the MoC subgroups included a majority of articles reporting on RCTs or cohort studies, i.e., stronger evidence (https://www.cebm.ox.ac.uk, *Intensive Case Management* MoC-−8 of 11; *Integrated Community Treatment* MoC-−6 of 10; see Table 3). The absence of good-quality study designs was evident in MoCs that were both longstanding (e.g., *rehabilitation and recovery-focused, supported accommodation*) and newer (*social and community connection-focused*, and *goal-focused*). Well-established models such as *intensive case management, vocational support*, and *supported accommodation* may be difficult to evaluate through RCTs as 'TAU' is not considered an ethical comparison (10). Perhaps this explains why a substantial minority of included studies examined subtypes of the model [e.g., (75)], newer modes of delivery [e.g., (49)], the relevance of the model to specific consumer subgroups [e.g., (51)], or enhancements to the model [e.g., (84)]. This methodological range made it difficult to conclude the overall benefits of each model, in relation to our outcomes. We also noted a lack of qualitative (*n* = 10) and mixed-method (*n* = 3) study designs. Qualitative research could provide a greater understanding of each model's benefits and how to maximize their value to consumers, while also soliciting their perspectives.

Some high-quality studies failed to show benefits to consumers in relation to our outcomes of interest. For example, three studies of *intensive case management* failed to show improved consumer self-efficacy (46, 48, 49). Two trials of staff training in comprehensive approaches to rehabilitation (63, 77) were negative. Three trials of enhancements for vocational services were negative (81, 84, 85). In the *social and community connection-focused* model, there were null findings for functional and personal recovery of individually delivered peer supports (66, 69). Various explanations are reported, beyond the conclusion that the model is not effective, concerning the outcomes in question. The impact of comparing the model with an active control (63), difficulties in implementing rehabilitation-supporting practice (77), and low engagement in the model (66, 69, 81) may all be relevant and have been reported elsewhere (109).

There were further subtle differences in the quality of the evidence within sub-groupings of articles. For example, *integrated community treatment* models of care research activity generally scored quite high against the Kmet criteria, suggesting that recent trials have led to more consistent and robust evidence of the value of this model of care. *Intensive case management* had wider variation in the quality of the studies, with scores ranging from 10 to 95, suggesting that the research base for this MoC has developed less consistently, resulting in a more mixed picture concerning study quality.

Bearing these issues in mind, our findings provided promising evidence for clinical and functional outcomes associated with *intensive case management, rehabilitation and recovery-focused*, and *integrated community treatment* models, with the latter also showing some promise for supporting personal recovery. Nonetheless, these findings are consistent with previous calls for more high-quality research concerning such models (18, 110), especially studies of outcomes valued by consumers (and their families) themselves (111). *Supported accommodation* and models focused on goals or social and community connections are all emerging practices with respect to supporting personal recovery. Models focused on specific outcomes consistent with consumers' priorities also appear helpful in supporting functional recovery. Although the potential for *vocational support* to support personal recovery is well-recognized (112), we found limited evidence for improved personal and functional recovery. A specific care coordination service model for people living with severe and persistent mental illness showed emerging evidence of benefits for personal recovery (*Partners in recovery care coordination*). This is noteworthy, since such approaches offer the potential to overcome recognized service access difficulties experienced by this group of consumers (113).

## Limitations of the included studies

The included studies had some limitations. There were far more quantitative studies than qualitative, but the quality of these studies was not consistently strong. Across all included studies where there was capacity for blinding, this was not typically used. The studies were often small-scale, lacked a comparison group, had high drop-out rates, and did not report on the sustainability of changes longitudinally, or on between-group differences. Studies had potential response bias, and some had difficulties in sustaining the engagement of participants (40, 66). Another limitation is the time frame we chose. There are potentially relevant studies that predated our review period.

## Discussion

Our review considered the recovery outcomes achieved by both long-standing and emerging models of care in mental health service delivery. Our purpose was to consider the attention being given to recovery outcomes, considering the overarching imperative to ensure models of care are addressing what people want and need. In previous research, consumers have been asked what they consider to be the most important issues in their lives. Most of these priorities are related to clinical, functional, and personal recovery, including social connection and managing distressing symptoms (5).

There are significant expectations that mental health services can demonstrate the implementation of MoCs, especially for people with persistent and complex mental health needs. This review originated in efforts to identify optimal MoCs in the context of a potentially transformative process being undertaken by the Royal Commission into Victoria Mental Health Services (RCVMHS) (10). Model of care is a term frequently used and perhaps relied on for system development and reform, but this review confirmed there is minimal consistency regarding its meaning.

We concluded that community-based MoCs include a delivery component that defines how care is provided and a content component which comprises a set of interventions. In our previous study, we demonstrated that MoCs are usually delivered within a multidisciplinary team that increasingly includes peer support workers or experts through Lived Experience (10). While we established a definition related to providing a holistic approach to treatment, care, and support, it was difficult to apply our definition in practice. We found that defining and grouping MoCs required careful thought and agreement among our team. We noticed some MoCs were well-established, such as *intensive case management, supported accommodation*, and *vocational support*, while others were emerging. The evolution of the models impacted the quality of evidence, the outcomes that tended to focus on, and the degree to which they were incorporating more contemporary paradigms and practices. For example, there was variation in the degree to which personal recovery and peer support were incorporated into service provision and research. Some MoCs, particularly the more well-established, had good detail about structure, consumer group, and content, whereas newer MoCs tended to be less well-defined and consistent.

The diversity of content and delivery components within related MoCs may represent innovation and local adaptation on the one hand [e.g., (114)] and model drift and poor implementation on the other. Alternatively, it may represent a tailoring to the needs of a cohort that becomes better understood over time. This is the crux of the challenge in implementing any complex intervention, of which any MoC is a prime example (115). Indeed, a focus on fidelity may unintentionally restrict how an MoC might adapt in relation to local contexts and fluctuating consumer needs, emergent knowledge, or in relation to the way outcomes are measured. For example, in this review, many v*ocational support* MoCs were excluded because the predominant primary outcome measure in these studies remains the number of days or hours a participant was in paid employment—not an individual recovery outcome measure. Although we cannot conclude this from our review, it is feasible that MoCs that do not evolve to become more person-centered and adopt contemporary ideas about what represents a “good” outcome for consumers, may be at risk of reinforcing more “institutional” practices, especially when services are under pressure. However, we noted that even MoCs achieving positive recovery outcomes (55–57) are not guaranteed ongoing support to enable them to consolidate, as is evident in the *partners in recovery care coordination* model, which has been gradually defunded in Australia, despite the promising evidence (116).

One sign of the evolution of a model is the incorporation of Lived Experience perspectives and peer support workers. This is gaining traction, but it requires evaluation of why and how it works, how it adds value, and when and how it is appropriately included. Overall, consumer input could have been used more widely to increase understanding of the research problem, co-develop and co-conduct the research, and improve data collection and dissemination (117). People with persistent and complex mental health needs commonly experience marginalization and exclusion, including opportunities to participate in research and service improvement. Ensuring their voices are heard is an opportunity to address this gap (118).

The lack of attention to recovery outcomes may be related to the timeframes studies are conducted within—it may take many years to see genuine recovery outcomes, and recovery tends not to be linear. So, even studies with a 1- or 2-year follow-up (and rarely it is longer) may find measuring recovery unrealistic—especially personal recovery (119). There are also issues in relation to whether the “dose” participants received in the timeframe of a study is sufficient to measure its impact (80). The individual meaning of personal recovery may create measurement challenges, but similar complexity is dealt with elsewhere, e.g., in cognitive neuroscience where no one person has the same pattern of individual strengths and weaknesses (120)—so it ought to be possible to incorporate recovery measures that respond to this challenge, especially if the goal of MoCs is be person-centered, holistic, and recovery-oriented (121).

Evaluation of models of care can be challenging when uptake and engagement are low [e.g., (42, 85, 87)]. This again suggests the importance of appreciating the context of intervention and the factors explaining poor engagement, or high levels of attrition (34). The impact of COVID-19 on attrition and completion rates needs to also be considered as this potentially had an important impact on studies [e.g., (37)]. Funding uncertainty also appears to impact outcomes (57).

## Conclusion and recommendations

Our findings provided promising and emerging evidence for recovery outcomes associated with a range of existing models of care. However, MoCs should evolve to meet the varied needs of people with persistent and complex mental health problems. Our review suggests the need for a sophisticated response that likely requires a multidisciplinary team approach, to respond and adapt to the context within which a MoC operates. While MoCs need specific design parameters for successful implementation, they also need to be able to incorporate consumer perspectives, ideally through co-design, to enhance recovery outcomes (122). Therefore, we recommend that (1) further research be conducted into outcomes associated with the MoCs we identified, ensuring that all three types of outcomes are assessed; (2) future research into these models should prioritize a consistent set of reliable and valid outcome measures such as the RAS and MANSA and attend to other identified methodological limitations so that meta-analyses can be conducted; (3) practice guidelines should recommend team-based models of care in line with this evidence, such as ICM and ICT, while enhancements to team-based MoCs should particularly focus on supporting personal recovery; (4) when developing new service models and improving existing service models for this consumer group, consumer goals and priorities should be a specific focus, to help clinicians challenge unhelpful ways of relating to consumers, and (5) further research should attend more closely to timeframes, including extended follow-up phases, to understand recovery impacts over time.

## Data availability statement

The original contributions presented in the study are included in the article/supplementary material, further inquiries can be directed to the corresponding author.

## Author contributions

CH: Conceptualization, Data curation, Formal analysis, Investigation, Methodology, Project administration, Resources, Supervision, Writing—original draft, Writing—review and editing. T-MZ: Data curation, Formal analysis, Investigation, Project administration, Writing—original draft, Writing—review and editing. CB: Data curation, Formal analysis, Investigation, Methodology, Writing—original draft, Writing—review and editing. PE: Data curation, Formal analysis, Investigation, Methodology, Writing—original draft, Writing—review and editing. JF: Conceptualization, Data curation, Formal analysis, Investigation, Methodology, Writing—original draft, Writing—review and editing. BH: Data curation, Formal analysis, Investigation, Methodology, Supervision, Writing—original draft, Writing—review and editing. HKi: Data curation, Formal analysis, Investigation, Methodology, Writing—original draft, Writing—review and editing. PM: Formal analysis, Investigation, Writing—review and editing. HKe: Data curation, Formal analysis, Writing—review and editing. LB: Conceptualization, Data curation, Formal analysis, Investigation, Methodology, Project administration, Resources, Supervision, Writing—original draft, Writing—review and editing.
